# Supplementary material for: The anti-symmetric and anisotropic symmetric exchange interactions between electric dipoles in hafnia
Source: Nat Commun. 2023 Dec 8;14:8127. doi: 10.1038/s41467-023-43593-5 (PMC10709352; doi:10.1038/s41467-023-43593-5)
Supplement: Supplementary file 1 — Supplementary Information [file 41467_2023_43593_MOESM1_ESM.pdf]

Supplementary Information for
"The anti-symmetric and anisotropic symmetric exchange interactions between electric dipoles in hafnia"

Longju Yu,[1] Hong Jian Zhao,[1,2,3,4,*] Peng Chen,[5] Laurent Bellaiche,[5] and Yanming Ma[1,3,4,†]

[1] *Key Laboratory of Material Simulation Methods and Software of Ministry of Education, College of Physics, Jilin University, Changchun 130012, China*
[2] *Key Laboratory of Physics and Technology for Advanced Batteries (Ministry of Education), College of Physics, Jilin University, Changchun 130012, China*
[3] *State Key Laboratory of Superhard Materials, College of Physics, Jilin University, Changchun 130012, China*
[4] *International Center of Future Science, Jilin University, Changchun 130012, China*
[5] *Physics Department and Institute for Nanoscience and Engineering, University of Arkansas, Fayetteville, Arkansas 72701, USA*

This Supplementary Information contains six Supplementary Notes, nine Supplementary Figures, twenty-two Supplementary Tables and Supplementary References, summarized as follows.

-Supplementary Note 1 – Symmetry analysis
-Supplementary Note 2 – Various phases of HfO_2 with non-collinear dipole patterns
-Supplementary Note 3 – The exchange interactions contributed by the O_{γ}^{W}-type distortion
-Supplementary Note 4 – The revisitation of NCDP in hafnia
-Supplementary Note 5 – Other possible mechanisms for the anti-symmetric exchange interactions
-Supplementary Note 6 – The symmetry rules for the exchange interactions between electric dipoles
-Supplementary Fig. 1 – Numerical verification of various trilinear couplings in H_l ($l = 5 - 11$)
-Supplementary Fig. 2 – Structural distortions in the $P2_1/c$ phase of HfO_2 oxide
-Supplementary Fig. 3 – Structural distortions in the $Pmn2_1$ phase of HfO_2 oxide
-Supplementary Fig. 4 – Structural distortions in the $Pca2_1$ phase of HfO_2 oxide
-Supplementary Fig. 5 – Structural distortions in the $Pbca$ phase of HfO_2 oxide
-Supplementary Fig. 6 – The lattice modes associated with the Hf sublattice
-Supplementary Fig. 7 – Several typical order parameters accommodated by the supercell of hafnia
-Supplementary Fig. 8 – Numerical verification of the trilinear coupling in H_{sp}
-Supplementary Fig. 9 – The coupling between u_i and u_j dipoles
-Supplementary Table 1 – The transformation rules of various order parameters for HfO_2 under the generators of the $Fm\bar{3}m$ space group
-Supplementary Tables 2, 4, 6, 8 – The anti-symmetric exchange interaction $A'_{\tau\kappa}$ between the dipoles centered on $\mathbf{R}_m + \mathbf{r}_\tau$ and $\mathbf{R}_m + \mathbf{r}_\kappa$
-Supplementary Tables 3, 5, 7, 9-16 – The anisotropic symmetric exchange interaction $S'_{\tau\kappa}$ between the dipoles centered on $\mathbf{R}_m + \mathbf{r}_\tau$ and $\mathbf{R}_m + \mathbf{r}_\kappa$
-Supplementary Tables 17-20 – The $A'_{\tau\kappa}$ and $S'_{\tau\kappa}$ exchange interactions in hafnia
-Supplementary Table 21 – Seven spatial symmetry operations
-Supplementary Table 22 – The symmetry rules regarding the anti-symmetric and symmetric exchange interactions between electric dipoles
-Supplementary References

* physzhaohj@jlu.edu.cn
† mym@jlu.edu.cn

SUPPLEMENTARY NOTE 1. SYMMETRY ANALYSIS

To begin with, we select the cubic $Fm\bar{3}m$ phase of HfO_2 as our high-symmetric reference phase. The conventional cell of $Fm\bar{3}m$ HfO_2 contains four formula units. In total, the motions of four Hf atoms and eight O atoms constitute thirty-six degrees of freedom (DOFs). These thirty-six DOFs can further be identified as thirty-six structural order parameters defined in Supplementary Table 1 and sketched in Fig. 1 of the Main Text. The thirty-six order parameters are preliminary classified into nine groups: (i) Hf_x^F, Hf_y^F, Hf_z^F; (ii) Hf_x^X, Hf_y^Y, Hf_z^Z; (iii) Hf_y^X, Hf_z^X, Hf_x^Y, Hf_z^Y, Hf_x^Z, Hf_y^Z; (iv) O_x^F, O_y^F, O_z^F; (v) O_x^G, O_y^G, O_z^G; (vi) $O_x^{A_x}$, $O_y^{A_y}$, $O_z^{A_z}$; (vii) $O_y^{A_x}$, $O_z^{A_x}$, $O_x^{A_y}$, $O_z^{A_y}$, $O_x^{A_z}$, $O_y^{A_z}$; (viii) $O_x^{C_x}$, $O_y^{C_y}$, $O_z^{C_z}$; and (ix) $O_y^{C_x}$, $O_z^{C_x}$, $O_x^{C_y}$, $O_z^{C_y}$, $O_x^{C_z}$, $O_y^{C_z}$, where each group of order parameters form an invariant subspace with respect to the symmetry operations in $Fm\bar{3}m$ phase.

Our next target is to derive the symmetry-allowed energetic couplings involving these thirty-six order parameters. In the present work, we are particularly interested in the trilinear couplings having the form of $Hf_\alpha^U Hf_\beta^V O_\gamma^W$. Here, $\alpha, \beta, \gamma = x, y, z$ denote the Cartesian component of each order parameter; $U, V = F, X, Y, Z$, and $W = F, G, A_x, A_y, A_z, C_x, C_y, C_z$ define the configuration that describes the motions of Hf or O ions (see Fig. 1 of the Main Text). Before deriving the symmetry-allowed couplings, we examine the transformation rules of these order parameters (see Supplementary Table 1) under the generators of the $Fm\bar{3}m$ space group. (Note that the symmetry operations of a specific group can be created by the generators of that group. The generators of each space group can be found at `https://www.cryst.ehu.es/cryst/get_gen.html`.) We recall that our desired $Hf_\alpha^U Hf_\beta^V O_\gamma^W$-type energetic couplings should be invariant under these generators (see Supplementary Table 1).

In the following, we will determine the $Hf_\alpha^U Hf_\beta^V O_\gamma^W$ couplings that are invariant under 2_{001}, 2_{010}, 3^+_{111}, 2_{110}, $\bar{1}$, $t(0,1/2,1/2)$ and $t(1/2,0,1/2)$ symmetry operations (see the caption of Supplementary Table 1 for the definition of these operations). Now we demonstrate how to derive the symmetry-allowed $Hf_\alpha^U Hf_\beta^V O_\gamma^W$ couplings. As an example, we choose Hf_α^U from (Hf_x^F, Hf_y^F, Hf_z^F), and Hf_β^V from (Hf_x^X, Hf_y^Y, Hf_z^Z). For simplicity, we further assume that $\alpha = x$ and $\beta = y$. The $Hf_\alpha^U Hf_\beta^V = Hf_x^F Hf_y^Y$ complies with the following transformation rules, namely, $2_{001} : Hf_x^F Hf_y^Y \rightarrow Hf_x^F Hf_y^Y$, $2_{010} : Hf_x^F Hf_y^Y \rightarrow -Hf_x^F Hf_y^Y$, $\bar{1} : Hf_x^F Hf_y^Y \rightarrow Hf_x^F Hf_y^Y$, $t(0,1/2,1/2) : Hf_x^F Hf_y^Y \rightarrow -Hf_x^F Hf_y^Y$, and $t(1/2,0,1/2) : Hf_x^F Hf_y^Y \rightarrow Hf_x^F Hf_y^Y$. In other words, $Hf_x^F Hf_y^Y$ is invariant under 2_{001}, $\bar{1}$, and $t(1/2,0,1/2)$ symmetry operations, but gain a "minus" sign under 2_{010} and $t(0,1/2,1/2)$ operations. Searching from Supplementary Table 1, the order parameter $O_x^{A_y}$ transform identically as $Hf_x^F Hf_y^Y$, under 2_{001}, 2_{010}, $\bar{1}$, $t(0,1/2,1/2)$, and $t(1/2,0,1/2)$ symmetry operations. This indicates that $Hf_x^F Hf_y^Y O_x^{A_y}$ is invariant under these five symmetry operations. Operating 3^+_{111} once and twice to $Hf_x^F Hf_y^Y O_x^{A_y}$ results in $Hf_y^F Hf_z^Z O_y^{A_z}$ and $Hf_z^F Hf_x^X O_z^{A_x}$, respectively. Consequently, $Hf_x^F Hf_y^Y O_x^{A_y} + Hf_y^F Hf_z^Z O_y^{A_z} + Hf_z^F Hf_x^X O_z^{A_x}$ is invariant under 3^+_{111} together with 2_{001}, 2_{010}, $\bar{1}$, $t(0,1/2,1/2)$, and $t(1/2,0,1/2)$ operations. Similarly, 2_{110} further "expand" $Hf_x^F Hf_y^Y O_x^{A_y} + Hf_y^F Hf_z^Z O_y^{A_z} + Hf_z^F Hf_x^X O_z^{A_x}$ to $Hf_x^F Hf_y^Y O_x^{A_y} + Hf_y^F Hf_z^Z O_y^{A_z} + Hf_z^F Hf_x^X O_z^{A_x} + Hf_y^F Hf_x^X O_y^{A_x} + Hf_x^F Hf_z^Z O_x^{A_z} + Hf_z^F Hf_y^Y O_z^{A_y}$, which is invariant under the generators (and thus all the symmetry operations) of the $Fm\bar{3}m$ space group.

In the present work, we derive the symmetry-allowed trilinear couplings by the projection operator approach – a well-established technique in group theory [1] – the basic idea of which is demonstrated in the previous paragraph. By the projection operator technique, we obtain eleven $Hf_\alpha^U Hf_\beta^V O_\gamma^W$-type energetic couplings (effective Hamiltonians), given by

$$
\begin{aligned}
H_1 &= K_1(Hf_y^Y Hf_x^Z O_y^{A_x} + Hf_x^Y Hf_z^Z O_z^{A_x} + Hf_x^X Hf_y^Z O_x^{A_y} + Hf_y^X Hf_z^Z O_z^{A_y} + Hf_x^X Hf_z^Y O_x^{A_z} + Hf_z^X Hf_y^Y O_y^{A_z}), \\
H_2 &= K_2(Hf_y^F Hf_x^X O_y^{A_x} + Hf_z^F Hf_x^X O_z^{A_x} + Hf_x^F Hf_y^Y O_x^{A_y} + Hf_z^F Hf_y^Y O_z^{A_y} + Hf_x^F Hf_z^Z O_x^{A_z} + Hf_y^F Hf_z^Z O_y^{A_z}), \\
H_3 &= K_3(Hf_x^F Hf_y^X O_y^{A_x} + Hf_x^F Hf_z^X O_z^{A_x} + Hf_y^F Hf_x^Y O_x^{A_y} + Hf_y^F Hf_z^Y O_z^{A_y} + Hf_z^F Hf_x^Z O_x^{A_z} + Hf_z^F Hf_y^Z O_y^{A_z}), \\
H_4 &= K_4(Hf_x^Y Hf_y^Z O_y^{A_x} + Hf_z^Y Hf_x^Z O_z^{A_x} + Hf_y^X Hf_x^Z O_x^{A_y} + Hf_z^X Hf_y^Z O_z^{A_y} + Hf_z^X Hf_x^Y O_x^{A_z} + Hf_y^X Hf_z^Y O_y^{A_z}), \\
H_5 &= K_5(Hf_y^F Hf_z^F O_x^G + Hf_x^F Hf_z^F O_y^G + Hf_x^F Hf_y^F O_z^G), \\
H_6 &= K_6(Hf_y^X Hf_z^X O_x^G + Hf_x^Y Hf_z^Y O_y^G + Hf_x^Z Hf_y^Z O_z^G), \qquad (1) \\
H_7 &= K_7(Hf_y^Y Hf_z^Y O_x^G + Hf_y^Z Hf_z^Z O_x^G + Hf_x^X Hf_z^X O_y^G + Hf_x^Z Hf_z^Z O_y^G + Hf_x^X Hf_y^X O_z^G + Hf_x^Y Hf_y^Y O_z^G), \\
H_8 &= K_8(Hf_x^Y Hf_x^Z O_x^{A_x} + Hf_y^X Hf_y^Z O_y^{A_y} + Hf_z^X Hf_z^Y O_z^{A_z}),
\end{aligned}
$$

$$H_9 = K_9(Hf_x^F Hf_x^X O_x^{A_x} + Hf_y^F Hf_y^Y O_y^{A_y} + Hf_z^F Hf_z^Z O_z^{A_z}),$$
$$H_{10} = K_{10}(Hf_y^Y Hf_y^Z O_x^{A_x} + Hf_z^Y Hf_z^Z O_x^{A_x} + Hf_x^X Hf_x^Z O_y^{A_y} + Hf_z^X Hf_z^Z O_y^{A_y} + Hf_x^X Hf_x^Y O_z^{A_z} + Hf_y^X Hf_y^Y O_z^{A_z}),$$
$$H_{11} = K_{11}(Hf_y^F Hf_y^X O_x^{A_x} + Hf_z^F Hf_z^X O_x^{A_x} + Hf_x^F Hf_x^Y O_y^{A_y} + Hf_z^F Hf_z^Y O_y^{A_y} + Hf_x^F Hf_x^Z O_z^{A_z} + Hf_y^F Hf_y^Z O_z^{A_z}),$$

where K_l ($l = 1 - 11$) are coefficients. Among them, H_l ($l = 1 - 4$) are of the form $Hf_\alpha^U Hf_\beta^V O_\gamma^W$ ($U \neq V$, $\alpha \neq \beta$). These couplings can interpret the non-collinear dipole patterns (NCDP) in $P2_1/c$, $Pmn2_1$, $Pca2_1$ and $Pbca$ phases of HfO_2 (see Supplementary Note 2 for the details). In contrast, H_l ($l = 5 - 7$) and H_l ($l = 8 - 11$) are effective Hamiltonians with the types of $Hf_\alpha^U Hf_\beta^U O_\gamma^W$ ($\alpha \neq \beta$) and $Hf_\alpha^U Hf_\alpha^V O_\gamma^W$ ($U \neq V$), respectively, being irrelevant to the NCDP. In Fig. 2 of the Main Text and Supplementary Fig. 1, we numerically verify various trilinear couplings in H_l ($l = 1 - 11$).

SUPPLEMENTARY NOTE 2. VARIOUS PHASES OF HfO_2 WITH NON-COLLINEAR DIPOLE PATTERNS

The HfO_2 oxide presents various phases that were discovered experimentally or predicted theoretically [2–12]. Of particular interest are the $P2_1/c$, $Pmn2_1$, $Pca2_1$ and $Pbca$ phases which showcase NCDP. In the following, we analyze the atomic motions for $P2_1/c$, $Pmn2_1$, $Pca2_1$ and $Pbca$ phases by the AMPLIMODES and ISODISTORT toolkits which can be found at `https://www.cryst.ehu.es/cryst/amplimodes.html` and `https://stokes.byu.edu/iso/isodistort.php`, respectively.

A. $P2_1/c$ phase

Supplementary Fig. 2 sketches the atomic motions for $P2_1/c$ phase of HfO_2, relative to $Fm\bar{3}m$. The $P2_1/c$ phase contains Hf_y^Z, Hf_z^X and Hf_x^X order parameters associated with the Hf sublattice. Apart from that, the O sublattice presents various distortion modes labelled by O_y^G, $O_x^{C_z}$, $O_z^{C_z}$, $O_y^{C_x}$, $O_x^{A_y}$ and $O_z^{A_y}$, respectively. Here, the combination of Hf_x^X and Hf_z^X, resulting from $Hf_x^X Hf_z^X O_y^G$ coupling [see H_7 in Supplementary Equation (1)], gives rise to collinear atomic motions of Hf. In contrast, (Hf_y^Z, Hf_z^X) and (Hf_y^Z, Hf_x^X) combinations lead to non-collinear atomic motions. The NCDP linked with (Hf_y^Z, Hf_z^X) and (Hf_y^Z, Hf_x^X) combinations are rooted in the $Hf_x^X Hf_y^Z O_x^{A_y}$ and $Hf_z^X Hf_y^Z O_z^{A_y}$ trilinear coupling terms [see H_1 and H_4 in Supplementary Equation (1)], respectively. In such sense, the $O_x^{A_y}$ and $O_z^{A_y}$ distortion modes provide the structural origin for the NCDP in $P2_1/c$ phase of HfO_2.

B. $Pmn2_1$ phase

The distortion modes for $Pmn2_1$ phase of HfO_2 are sketched in Supplementary Fig. 3. With respect to $Fm\bar{3}m$ phase, $Pmn2_1$ phase contains the following order parameters: Hf_y^Y, Hf_z^F, Hf_x^F, O_y^G, O_x^F, O_z^F, $O_y^{C_y}$, $O_x^{A_y}$, and $O_z^{A_y}$. The (Hf_x^F, Hf_z^F) combination suggested by $Hf_x^F Hf_z^F O_y^G$ coupling [see H_5 in Supplementary Equation (1)] yields collinear atomic motions of Hf. The non-collinear motions arise from the other combinations (Hf_y^Y, Hf_z^F) and (Hf_y^Y, Hf_x^F) . Such non-collinear motions can be interpreted by $Hf_z^F Hf_y^Y O_z^{A_y}$ and $Hf_x^F Hf_y^Y O_x^{A_y}$ couplings [shown in H_2 of Supplementary Equation (1)]. This indicates that the NCDP in $Pmn2_1$ phase are rooted in $O_x^{A_y}$ and $O_z^{A_y}$ distortion modes.

C. $Pca2_1$ phase

In Supplementary Fig. 4, we show the sketches of various atomic motions for $Pca2_1$ phase of HfO_2. The structural distortion modes of $Pca2_1$ phase (with respect to $Fm\bar{3}m$ phase) can be decomposed into Hf_y^Y, Hf_z^F, Hf_x^Z, O_z^F, $O_y^{C_z}$, $O_x^{A_z}$, $O_x^{C_x}$, $O_y^{A_x}$, and $O_z^{A_y}$. In the $Pca2_1$ phase, there are three $(Hf_\alpha^U, Hf_\beta^V)$ combinations that can lead to non-collinear motions of Hf ions. These combinations are (Hf_y^Y, Hf_x^Z), (Hf_y^Y, Hf_z^F) and (Hf_z^F, Hf_x^Z), arising from $Hf_y^Y Hf_x^Z O_y^{A_x}$, $Hf_z^F Hf_y^Y O_z^{A_y}$ and $Hf_z^F Hf_x^Z O_x^{A_z}$ couplings, respectively [see H_1, H_2 and H_3 in Supplementary Equation (1)]. The NCDP in $Pca2_1$ are rooted in $O_y^{A_x}$, $O_z^{A_y}$ and $O_x^{A_z}$ structural distortions.

D. $Pbca$ phase

As shown in Supplementary Fig. 5, the atomic motions for the $Pbca$ phase of HfO_2 are more complicated than those for $P2_1/c$, $Pmn2_1$ and $Pca2_1$ phases. The structural distortions of $Pbca$ not only involve the simple modes [see Supplementary Figs. **5a**-**5d**] defined within the $Fm\bar{3}m$ cell, but also present complex modes [see Supplementary Figs. **5e**-**5h**] hosting which requires doubling the cell with respect to the conventional cell of $Fm\bar{3}m$. As a result,

the atomic motions sketched in Supplementary Figs. 5**a**-5**d** can be described within our framework demonstrated in Fig. 1 of the Main Text. These modes are thus assigned to symbols such as $O_y^{A_z}$ and Hf_z^X. In contrast, the atomic motions shown in Supplementary Figs. 5**e**-5**h** can not be linked with our symbols defined in Fig. 1 of the Main Text.

Note that, combining Hf_y^Y (or Hf_z^X) with the modes sketched in Supplementary Fig. 5**h** does yield NCDP in *Pbca* phase. However, describing such behaviors requires the definition of more complicated order parameters, which is beyond the scope of the present work. We thus discard the discussion related to the order parameters shown in Supplementary Fig. 5**h**. In *Pbca*, we are interested in the trilinear couplings incorporating Hf_y^Y and Hf_z^X order parameters. Such a coupling is identified as $Hf_z^X Hf_y^Y O_y^{A_z}$ [see H_1 in Supplementary Equation (1)], implying that $O_y^{A_z}$ distortion yields NCDP in the *Pbca* phase.

SUPPLEMENTARY NOTE 3. THE EXCHANGE INTERACTIONS CONTRIBUTED BY THE O_γ^W-TYPE DISTORTION

We now unravel the exchange interaction between dipoles (centered on Hf ions) that are hosted by our aforementioned energetic couplings H_l ($l = 1-11$). To this end, we represent each order parameter Hf_α^U by an atomic basis. We start from the conventional cell of $Fm\bar{3}m$ HfO_2. With respect to this cell, the coordinates of Hf ions are given by $\mathbf{r}_1 = 0$, $\mathbf{r}_2 = \frac{1}{2}\mathbf{b} + \frac{1}{2}\mathbf{c}$, $\mathbf{r}_3 = \frac{1}{2}\mathbf{a} + \frac{1}{2}\mathbf{c}$ and $\mathbf{r}_4 = \frac{1}{2}\mathbf{a} + \frac{1}{2}\mathbf{b}$ (**a**, **b**, **c** being the lattice vectors). We then work with a big supercell containing N conventional cells. As a consequence, the coordinate of each Hf ion within the supercell is given by $\mathbf{R}_m + \mathbf{r}_\tau$, where m labels the m_{th} conventional cell inside the supercell and τ labels the τ_{th} Hf ion in the m_{th} cell ($m = 1, 2, ..., N$; $\tau = 1, 2, 3, 4$); In total, there are $4N$ Hf ions in the supercell. By structural distortion, each Hf ion gains a displacement $u_{m,\tau,\alpha}$ relative to $\mathbf{R}_m + \mathbf{r}_\tau$ with $\alpha(= x, y, z)$ denoting the direction of that displacement. According to Supplementary Fig. 6, the order parameters Hf_α^F, Hf_α^X, Hf_α^Y, and Hf_α^Z can be written as

$$\begin{aligned}
Hf_\alpha^F &= \frac{1}{4N}\sum_m (u_{m,1,\alpha} + u_{m,2,\alpha} + u_{m,3,\alpha} + u_{m,4,\alpha}),\\
Hf_\alpha^X &= \frac{1}{4N}\sum_m (u_{m,1,\alpha} + u_{m,2,\alpha} - u_{m,3,\alpha} - u_{m,4,\alpha}),\\
Hf_\alpha^Y &= \frac{1}{4N}\sum_m (u_{m,1,\alpha} - u_{m,2,\alpha} + u_{m,3,\alpha} - u_{m,4,\alpha}),\\
Hf_\alpha^Z &= \frac{1}{4N}\sum_m (u_{m,1,\alpha} - u_{m,2,\alpha} - u_{m,3,\alpha} + u_{m,4,\alpha}).
\end{aligned} \tag{2}$$

First of all, we focus on the exchange interactions that are connected with NCDP [hosted by H_l ($l = 1-4$)]. Inserting Supplementary Equation (2) into H_l ($l = 1-4$) yields the following expressions:

$$\begin{aligned}
H_1 \propto \sum_{mm'} [&(u_{m,1,y} - u_{m,2,y} + u_{m,3,y} - u_{m,4,y})(u_{m',1,x} - u_{m',2,x} - u_{m',3,x} + u_{m',4,x})O_y^{A_x}\\
&+(u_{m,1,x} - u_{m,2,x} + u_{m,3,x} - u_{m,4,x})(u_{m',1,z} - u_{m',2,z} - u_{m',3,z} + u_{m',4,z})O_z^{A_x}\\
&+(u_{m,1,x} + u_{m,2,x} - u_{m,3,x} - u_{m,4,x})(u_{m',1,y} - u_{m',2,y} - u_{m',3,y} + u_{m',4,y})O_x^{A_y}\\
&+(u_{m,1,y} + u_{m,2,y} - u_{m,3,y} - u_{m,4,y})(u_{m',1,z} - u_{m',2,z} - u_{m',3,z} + u_{m',4,z})O_z^{A_y}\\
&+(u_{m,1,x} + u_{m,2,x} - u_{m,3,x} - u_{m,4,x})(u_{m',1,z} - u_{m',2,z} + u_{m',3,z} - u_{m',4,z})O_x^{A_z}\\
&+(u_{m,1,z} + u_{m,2,z} - u_{m,3,z} - u_{m,4,z})(u_{m',1,y} - u_{m',2,y} + u_{m',3,y} - u_{m',4,y})O_y^{A_z}],
\end{aligned} \tag{3}$$

$$H_2 \propto \sum_{mm'} [(u_{m,1,y}+u_{m,2,y}+u_{m,3,y}+u_{m,4,y})(u_{m',1,x}+u_{m',2,x}-u_{m',3,x}-u_{m',4,x})O_y^{A_x}$$
$$+(u_{m,1,z}+u_{m,2,z}+u_{m,3,z}+u_{m,4,z})(u_{m',1,x}+u_{m',2,x}-u_{m',3,x}-u_{m',4,x})O_z^{A_x}$$
$$+(u_{m,1,x}+u_{m,2,x}+u_{m,3,x}+u_{m,4,x})(u_{m',1,y}-u_{m',2,y}+u_{m',3,y}-u_{m',4,y})O_x^{A_y} \quad (4)$$
$$+(u_{m,1,z}+u_{m,2,z}+u_{m,3,z}+u_{m,4,z})(u_{m',1,y}-u_{m',2,y}+u_{m',3,y}-u_{m',4,y})O_z^{A_y}$$
$$+(u_{m,1,x}+u_{m,2,x}+u_{m,3,x}+u_{m,4,x})(u_{m',1,z}-u_{m',2,z}-u_{m',3,z}+u_{m',4,z})O_x^{A_z}$$
$$+(u_{m,1,y}+u_{m,2,y}+u_{m,3,y}+u_{m,4,y})(u_{m',1,z}-u_{m',2,z}-u_{m',3,z}+u_{m',4,z})O_y^{A_z}],$$

$$H_3 \propto \sum_{mm'} [(u_{m,1,x}+u_{m,2,x}+u_{m,3,x}+u_{m,4,x})(u_{m',1,y}+u_{m',2,y}-u_{m',3,y}-u_{m',4,y})O_y^{A_x}$$
$$+(u_{m,1,x}+u_{m,2,x}+u_{m,3,x}+u_{m,4,x})(u_{m',1,z}+u_{m',2,z}-u_{m',3,z}-u_{m',4,z})O_z^{A_x}$$
$$+(u_{m,1,y}+u_{m,2,y}+u_{m,3,y}+u_{m,4,y})(u_{m',1,x}-u_{m',2,x}+u_{m',3,x}-u_{m',4,x})O_x^{A_y} \quad (5)$$
$$+(u_{m,1,y}+u_{m,2,y}+u_{m,3,y}+u_{m,4,y})(u_{m',1,z}-u_{m',2,z}+u_{m',3,z}-u_{m',4,z})O_z^{A_y}$$
$$+(u_{m,1,z}+u_{m,2,z}+u_{m,3,z}+u_{m,4,z})(u_{m',1,x}-u_{m',2,x}-u_{m',3,x}+u_{m',4,x})O_x^{A_z}$$
$$+(u_{m,1,z}+u_{m,2,z}+u_{m,3,z}+u_{m,4,z})(u_{m',1,y}-u_{m',2,y}-u_{m',3,y}+u_{m',4,y})O_y^{A_z}],$$

$$H_4 \propto \sum_{mm'} [(u_{m,1,x}-u_{m,2,x}+u_{m,3,x}-u_{m,4,x})(u_{m',1,y}-u_{m',2,y}-u_{m',3,y}+u_{m',4,y})O_y^{A_x}$$
$$+(u_{m,1,z}-u_{m,2,z}+u_{m,3,z}-u_{m,4,z})(u_{m',1,x}-u_{m',2,x}-u_{m',3,x}+u_{m',4,x})O_z^{A_x}$$
$$+(u_{m,1,y}+u_{m,2,y}-u_{m,3,y}-u_{m,4,y})(u_{m',1,x}-u_{m',2,x}-u_{m',3,x}+u_{m',4,x})O_x^{A_y} \quad (6)$$
$$+(u_{m,1,z}+u_{m,2,z}-u_{m,3,z}-u_{m,4,z})(u_{m',1,y}-u_{m',2,y}-u_{m',3,y}+u_{m',4,y})O_z^{A_y}$$
$$+(u_{m,1,z}+u_{m,2,z}-u_{m,3,z}-u_{m,4,z})(u_{m',1,x}-u_{m',2,x}+u_{m',3,x}-u_{m',4,x})O_x^{A_z}$$
$$+(u_{m,1,y}+u_{m,2,y}-u_{m,3,y}-u_{m,4,y})(u_{m',1,z}-u_{m',2,z}+u_{m',3,z}-u_{m',4,z})O_y^{A_z}],$$

where m and m' label the m_{th} and m'_{th} cells, respectively.

Supplementary Equations (3)-(6) enable the determination of the O_γ^W-contributed exchange interactions between two dipoles (e.g., depicted by $u_{m,\tau,\alpha}$ and $u_{m',\kappa,\beta}$) that are centered on Hf ions. In the present work, we are interested in the interactions of dipoles located within the same conventional cell [i.e., $m = m' = 1$]. In the following discussion, we will omit the cell labels m and m'. As a result, the exchange interaction $J'_{\tau\kappa,\alpha\beta}$ associated with H_l can be calculated by

$$J'_{\tau\kappa} = \begin{pmatrix} J'_{\tau\kappa,xx} & J'_{\tau\kappa,xy} & J'_{\tau\kappa,xz} \\ J'_{\tau\kappa,yx} & J'_{\tau\kappa,yy} & J'_{\tau\kappa,yz} \\ J'_{\tau\kappa,zx} & J'_{\tau\kappa,zy} & J'_{\tau\kappa,zz} \end{pmatrix} = \begin{pmatrix} \frac{\partial^2 H_l}{\partial u_{\tau,x}\partial u_{\kappa,x}} & \frac{\partial^2 H_l}{\partial u_{\tau,x}\partial u_{\kappa,y}} & \frac{\partial^2 H_l}{\partial u_{\tau,x}\partial u_{\kappa,z}} \\ \frac{\partial^2 H_l}{\partial u_{\tau,y}\partial u_{\kappa,x}} & \frac{\partial^2 H_l}{\partial u_{\tau,y}\partial u_{\kappa,y}} & \frac{\partial^2 H_l}{\partial u_{\tau,y}\partial u_{\kappa,z}} \\ \frac{\partial^2 H_l}{\partial u_{\tau,z}\partial u_{\kappa,x}} & \frac{\partial^2 H_l}{\partial u_{\tau,z}\partial u_{\kappa,y}} & \frac{\partial^2 H_l}{\partial u_{\tau,z}\partial u_{j,z}} \end{pmatrix}. \quad (7)$$

The nine $J'_{\tau\kappa,\alpha\beta}$ components form a second rank tensor $J'_{\tau\kappa}$ characterizing the interactions of $u_{m,\tau,\alpha}$ and $u_{m',\kappa,\beta}$ [see Supplementary Equation (7)]. This tensor has only off-diagonal elements being non-zero [because the terms in Supplementary Equations (3)-(6) have the form of $u_{m,\tau,\alpha}u_{m',\kappa,\beta}O_\gamma^W$ with $\alpha \neq \beta$], and can be further rewritten as a summation of the anti-symmetric tensor $A'_{\tau\kappa}$ and the symmetric tensor $S'_{\tau\kappa}$ as follows:.

$$A'_{\tau\kappa} = \frac{1}{2}\begin{pmatrix} 0 & \frac{\partial^2 H_l}{\partial u_{\tau,x}\partial u_{\kappa,y}} - \frac{\partial^2 H_l}{\partial u_{\tau,y}\partial u_{\kappa,x}} & \frac{\partial^2 H_l}{\partial u_{\tau,x}\partial u_{\kappa,z}} - \frac{\partial^2 H_l}{\partial u_{\tau,z}\partial u_{\kappa,x}} \\ \frac{\partial^2 H_l}{\partial u_{\tau,y}\partial u_{\kappa,x}} - \frac{\partial^2 H_l}{\partial u_{\tau,x}\partial u_{\kappa,y}} & 0 & \frac{\partial^2 H_l}{\partial u_{\tau,y}\partial u_{\kappa,z}} - \frac{\partial^2 H_l}{\partial u_{\tau,z}\partial u_{\kappa,y}} \\ \frac{\partial^2 H_l}{\partial u_{\tau,z}\partial u_{\kappa,x}} - \frac{\partial^2 H_l}{\partial u_{\tau,x}\partial u_{\kappa,z}} & \frac{\partial^2 H_l}{\partial u_{\tau,z}\partial u_{\kappa,y}} - \frac{\partial^2 H_l}{\partial u_{\tau,y}\partial u_{\kappa,z}} & 0 \end{pmatrix},$$
$$S'_{\tau\kappa} = \frac{1}{2}\begin{pmatrix} 0 & \frac{\partial^2 H_l}{\partial u_{\tau,x}\partial u_{\kappa,y}} + \frac{\partial^2 H_l}{\partial u_{\tau,y}\partial u_{\kappa,x}} & \frac{\partial^2 H_l}{\partial u_{\tau,x}\partial u_{\kappa,z}} + \frac{\partial^2 H_l}{\partial u_{\tau,z}\partial u_{\kappa,x}} \\ \frac{\partial^2 H_l}{\partial u_{\tau,y}\partial u_{\kappa,x}} + \frac{\partial^2 H_l}{\partial u_{\tau,x}\partial u_{\kappa,y}} & 0 & \frac{\partial^2 H_l}{\partial u_{\tau,y}\partial u_{\kappa,z}} + \frac{\partial^2 H_l}{\partial u_{\tau,z}\partial u_{\kappa,y}} \\ \frac{\partial^2 H_l}{\partial u_{\tau,z}\partial u_{\kappa,x}} + \frac{\partial^2 H_l}{\partial u_{\tau,x}\partial u_{\kappa,z}} & \frac{\partial^2 H_l}{\partial u_{\tau,z}\partial u_{\kappa,y}} + \frac{\partial^2 H_l}{\partial u_{\tau,y}\partial u_{\kappa,z}} & 0 \end{pmatrix}. \quad (8)$$

Here, the $A'_{\tau\kappa}$ and $S'_{\tau\kappa}$ tensors describe the electric Dzyaloshinskii-Moriya interaction and the electric anisotropic symmetric exchange interaction, respectively. Following Supplementary Equations (3)-(6) and using Supplementary Equation (8), we can derive the $A'_{\tau\kappa}$ and $S'_{\tau\kappa}$ tensors that are hosted by H_l $(l = 1-4)$. For example, the derivation of $A'_{12,xy}$ from H_1 is demonstrated as

$$A'_{12,xy} = \frac{1}{2}\left(\frac{\partial^2 H_1}{\partial u_{1,x}\partial u_{2,y}} - \frac{\partial^2 H_1}{\partial u_{1,y}\partial u_{2,x}}\right) \propto \frac{1}{2}(-O_y^{A_x} - O_x^{A_y} + O_y^{A_x} - O_x^{A_y}) = -O_x^{A_y}. \tag{9}$$

Our derived $A'_{\tau\kappa}$ and $S'_{\tau\kappa}$ tensors – on the basis of H_l $(l = 1-4)$ – are summarized in Supplementary Tables 2-9.

We move on to extract the exchange interactions of dipoles from H_l $(l = 5-11)$. In H_l $(l = 5-7)$, each H_l (i) leads to a $A'_{\tau\kappa}$ tensor whose elements are all zero, and (ii) yields a $S'_{\tau\kappa}$ tensor with the diagonal elements being zero. Using the strategy as indicated in Supplementary Equation (2) to Supplementary Equation (9), we calculate the $S'_{\tau\kappa}$ tensors (see Supplementary Tables 10-12) with respect to H_l $(l = 5-7)$. As for H_l $(l = 8-11)$, the $S'_{\tau\kappa}$ tensors contain diagonal elements, and should be calculated by $S'_{\tau\kappa,\alpha\beta} = \frac{1}{2}(J'_{\tau\kappa,\alpha\beta} + J'_{\tau\kappa,\beta\alpha}) - \frac{1}{3}\delta_{\alpha,\beta}(J'_{\tau\kappa,xx} + J'_{\tau\kappa,yy} + J'_{\tau\kappa,zz})$ according to Equation (7) of the Main Text. Our derived $S'_{\tau\kappa}$ tensors are shown in Supplementary Tables 13-16.

Before finishing Supplementary Note 3, let us point out the limitations of our theories. Readers may notice that the interaction between $u_{m,\tau,\alpha}$ and $u_{m',\kappa,\beta}$ (i) is determined by the collective distortions from the O sublattice, instead of those localized around the region defined by $\mathbf{R}_m + \mathbf{r}_\tau$ and $\mathbf{R}_{m'} + \mathbf{r}_\kappa$, and (ii) does not depend on the distance between m and m' cells (i.e., $|\mathbf{R}_m - \mathbf{R}_{m'}|$). Indeed, our theories seem to suggest the non-local feature of the exchange interaction between two dipoles. This arises from the fact that our structural order parameters are defined with respect to the conventional cell of HfO_2, which merely contains four formula units. This is not enough to capture the nature of locality for the exchange interactions. To fix such an issue, an avenue is to define the order parameters in a large enough cell containing the Hf ions located at $\mathbf{R}_m + \mathbf{r}_\tau$ and $\mathbf{R}_{m'} + \mathbf{r}_\kappa$. By doing so, the couplings involving more complicated order parameters [see e.g., Supplementary Figs. 5**e**-5**h**] can be derived, and the interaction between dipoles should gain a localized feature. We also refer interested readers to Ref. [13] for the corresponding discussion regarding the anti-symmetric exchange interaction of dipoles in perovskites (see Supplementary Discussion IV in its Supplementary Information).

SUPPLEMENTARY NOTE 4. THE REVISITATION OF NCDP IN HAFNIA

Previously, we have shown that the NCDP in the $P2_1/c$, $Pmn2_1$, $Pca2_1$ and $Pbca$ phases of HfO_2 can be described by H_1, H_2, H_3 and H_4 (see Table 1 and Fig. 3 of the Main Text). The NCDP in these phases are ascribed to the O_γ^W-type structural distortions – driving the anti-symmetric $A'_{\tau\kappa}$ and symmetric $S'_{\tau\kappa}$ exchange interactions between electric dipoles. In this Supplementary Note, we will analyze the correlation among the $A'_{\tau\kappa}$ interaction, the $S'_{\tau\kappa}$ interaction and the NCDP in HfO_2. To this end, we start from the effective Hamiltonian $H = \alpha H_1 + \beta H_2 + \gamma H_3 + \delta H_4$ and consider various O_γ^W-type distortions that are involved in $P2_1/c$, $Pmn2_1$, $Pca2_1$ and $Pbca$ phases.

A. The $A'_{\tau\kappa}$ and $S'_{\tau\kappa}$ exchange interactions driven by $O_x^{A_y}$

As shown in Table 1 of the Main Text, the $O_x^{A_y}$ distortion drives the NCDP in $P2_1/c$ and $Pmn2_1$ phases of HfO_2. Following the procedures mentioned in Supplementary Note 3, the $A'_{\tau\kappa}$ and $S'_{\tau\kappa}$ exchange interactions (contributed by $O_x^{A_y}$) are calculated (see Table 2 in the Main Text). Here, the non-null anti-symmetric $A'_{\tau\kappa,xy}$ [(τ,κ)=(1,2), (1,4), (2,1), (2,3), (3,2), (3,4), (4,1), and (4,3)] interactions coincide with the non-collinear alignment of dipoles (associated with their x and y components) centered on Hf_τ and Hf_κ sites (see Figs. 3**b** and 3**e**). In contrast, the non-null symmetric $S'_{\tau\kappa,xy}$ [(τ,κ)=(1,3), (3,1), (2,4), and (4,2)] interaction connect to the collinear (i.e., antiparallel or parallel) alignments of dipoles centered on Hf_τ and Hf_κ sites (see Figs. 3**b** and 3**e**).

B. The $A'_{\tau\kappa}$ and $S'_{\tau\kappa}$ exchange interactions driven by $O_y^{A_x}$

The $O_y^{A_x}$ drives the NCDP in $Pca2_1$ phase (see Table 1 of the Main Text). The $O_y^{A_x}$-contributed $A'_{\tau\kappa}$ and $S'_{\tau\kappa}$ are extracted and shown in Supplementary Table 17. The non-null anti-symmetric $A'_{\tau\kappa,xy}$ [(τ,κ)=(1,3), (1,4), (2,3), (2,4), (3,1), (3,2), (4,1), and (4,2)] interactions interpret the non-collinear alignments of dipoles (associated with their x and y components) centered on Hf_τ and Hf_κ sites (see Fig. 3**c**). On the other hand, the non-null $S'_{\tau\kappa,xy}$ [(τ,κ)=(1,2), (2,1), (3,4), and (4,3)] are linked with the collinear (i.e., antiparallel) alignments of dipoles centered on Hf_τ and Hf_κ

sites (see Fig. 3**c**).

C. The $A'_{\tau\kappa}$ and $S'_{\tau\kappa}$ exchange interactions driven by $O_y^{A_z}$

The $O_y^{A_z}$ corresponds to the NCDP in *Pbca* phase (see Table 1 of the Main Text). As shown in Supplementary Table 18, we calculate the $A'_{\tau\kappa}$ and $S'_{\tau\kappa}$ interactions that are driven by the $O_y^{A_z}$ distortion. The non-null anti-symmetric $A'_{\tau\kappa,yz}$ [(τ,κ)=(1,2), (1,3), (2,1), (2,4), (3,1), (3,4), (4,2), and (4,3)] interactions are consistent with the non-collinear alignment of dipoles (associated with their y and z components) centered on Hf_τ and Hf_κ sites (see Fig. 3**d**). The non-null symmetric $S'_{\tau\kappa,yz}$ [(τ,κ)=(1,4), (2,3), (3,2), and (4,1)] interactions have connections to the collinear (i.e., antiparallel) alignments of dipoles centered on Hf_τ and Hf_κ sites (see Fig. 3**d**).

D. The $A'_{\tau\kappa}$ and $S'_{\tau\kappa}$ exchange interactions driven by $O_z^{A_y}$

As shown in Table 1 of the Main Text, the $O_z^{A_y}$ drives the NCDP in $Pmn2_1$, $P2_1/c$ and $Pca2_1$ phases. Supplementary Table 19 shows the calculated $A'_{\tau\kappa}$ and $S'_{\tau\kappa}$ being originated from the $O_z^{A_y}$ distortion. The non-null $A'_{\tau\kappa,yz}$ [(τ,κ)=(1,2), (1,4), (2,1), (2,3), (3,2), (3,4), (4,1), and (4,3)] interactions are related to the non-collinear alignment of dipoles (associated with their y and z components) centered on Hf_τ and Hf_κ sites (see Figs. 3**f** and 3**h**). The non-null $S'_{\tau\kappa,yz}$ [(τ,κ)=(1,3), (2,4), (3,1), and (4,2)] interactions correspond to the collinear (i.e., parallel or antiparallel) alignments of dipoles centered on Hf_τ and Hf_κ sites (see Figs. 3**f** and 3**h**).

E. The $A'_{\tau\kappa}$ and $S'_{\tau\kappa}$ exchange interactions driven by $O_x^{A_z}$

The $O_x^{A_z}$ distortion drives the NCDP in $Pca2_1$ phase (see Table 1 of the Main Text). The $A'_{\tau\kappa}$ and $S'_{\tau\kappa}$ exchange interactions contributed by $O_x^{A_z}$ are shown in Supplementary Table 20. The non-null $A'_{\tau\kappa,xz}$ [(τ,κ)=(1,2), (1,3), (2,1), (2,4), (3,1), (3,4), (4,2), and (4,3)] interactions interpret the non-collinear dipoles alignments (associated with their x and z components) on Hf_τ and Hf_κ sites (see Fig. 3**g**). The non-null $S'_{\tau\kappa,xz}$ [(τ,κ)=(1,4), (2,3), (3,2), and (4,1)] interactions are associated with the collinear (i.e., parallel) alignments of dipoles centered on Hf_τ and Hf_κ sites (see Fig. 3**g**).

SUPPLEMENTARY NOTE 5. OTHER POSSIBLE MECHANISMS FOR THE ANTI-SYMMETRIC EXCHANGE INTERACTIONS

In this Supplementary Note, we show that complicated couplings can be achieved by employing a larger supercell of HfO_2 as our starting point. To demonstrate this, we consider a $2\times2\times2$ supercell of HfO_2 (with respect to its conventional cell). Within this supercell, more complex order parameters [for instance, Hf_α^{R1}, Hf_α^{R2}, Hf_α^{R3} and Hf_α^{R4} ($\alpha = x,y,z$)] are defined (see Supplementary Fig. 7). A specific coupling that involves these order parameters is given by

$$\begin{aligned} H_{sp} \propto & Hf_z^{R1}Hf_x^{R2}O_x^{A_y} + Hf_x^{R1}Hf_y^{R3}O_y^{A_z} + Hf_y^{R3}Hf_z^{R1}O_y^{A_x} + Hf_y^{R1}Hf_z^{R4}O_z^{A_x} + Hf_x^{R1}Hf_z^{R4}O_z^{A_y} + Hf_y^{R1}Hf_x^{R2}O_x^{A_z} - \\ & Hf_x^{R1}Hf_z^{R2}O_x^{A_y} - Hf_y^{R1}Hf_x^{R3}O_y^{A_z} - Hf_z^{R3}Hf_y^{R1}O_y^{A_x} - Hf_z^{R1}Hf_y^{R4}O_z^{A_x} - Hf_z^{R1}Hf_x^{R4}O_z^{A_y} - Hf_x^{R1}Hf_y^{R2}O_x^{A_z} + \\ & Hf_x^{R3}Hf_z^{R4}O_x^{A_y} + Hf_x^{R2}Hf_y^{R4}O_y^{A_z} + Hf_y^{R2}Hf_z^{R4}O_y^{A_x} + Hf_z^{R2}Hf_y^{R3}O_z^{A_x} + Hf_x^{R2}Hf_z^{R3}O_z^{A_y} + Hf_y^{R3}Hf_x^{R4}O_x^{A_z} - \\ & Hf_z^{R3}Hf_x^{R4}O_x^{A_y} - Hf_y^{R2}Hf_x^{R4}O_y^{A_z} - Hf_z^{R2}Hf_y^{R4}O_y^{A_x} - Hf_y^{R2}Hf_z^{R3}O_z^{A_x} - Hf_z^{R2}Hf_x^{R3}O_z^{A_y} - Hf_x^{R3}Hf_y^{R4}O_x^{A_z}. \end{aligned} \quad (10)$$

Here, the $Hf_x^{R1}Hf_z^{R2}O_x^{A_y}$ term is numerically verified by first-principles simulations (see Supplementary Fig. 8), using the following strategy. Starting from the $2\times2\times2$ supercell of the conventional cell, we displace the O ions according to the $O_x^{A_y}$ mode by a fixed value of 0.05 a_{fc}. Next, we displace the Hf ions following the Hf_x^{R1} mode by various values varying from -0.05 a_{fc} to 0.05 a_{fc} with a step of 0.01 a_{fc}. Finally, we do first-principles self-consistent calculations (no structural relaxations) for the distorted structures, measure the resulted forces associated with the Hf_z^{R2} mode, and plot the forces as a function of Hf_x^{R1} mode.

The anti-symmetric exchange interaction driven by $O_x^{A_y}$, being associated with Supplementary Equation (10), is implied by the $Hf_z^{R1}Hf_x^{R2}O_x^{A_y} - Hf_x^{R1}Hf_z^{R2}O_x^{A_y} + Hf_x^{R3}Hf_z^{R4}O_x^{A_y} - Hf_z^{R3}Hf_x^{R4}O_x^{A_y}$ term. For example, such a term results in the anti-symmetric exchange interaction between Hf_1 and Hf_2, given by $A'_{12,xz} = -A'_{12,zx} \propto -O_x^{A_y}$.

SUPPLEMENTARY NOTE 6. THE SYMMETRY RULES FOR THE EXCHANGE INTERACTIONS BETWEEN ELECTRIC DIPOLES

Now we derive the symmetry rules regarding the anti-symmetric and symmetric exchange interactions between electric dipoles. Note that these symmetry rules are not limited to hafnia, but rather widely applicable to other

variety of materials. As shown in Supplementary Fig. 9, we consider two electric dipoles $\mathbf{u}_i \equiv (u_{i,x}, u_{i,y}, u_{i,z})$ and $\mathbf{u}_j \equiv (u_{j,x}, u_{j,y}, u_{j,z})$ located at the i_{th} and j_{th} sites. To derive the symmetry rules of the interaction, we define seven spatial symmetry operations that are shown in Supplementary Table 21, following Refs. [14, 15].

A. The anti-symmetric exchange interaction between electric dipoles

We recall that the anti-symmetric exchange interaction between $\mathbf{u}_i \equiv (u_{i,x}, u_{i,y}, u_{i,z})$ and $\mathbf{u}_j \equiv (u_{j,x}, u_{j,y}, u_{j,z})$ electric dipoles can be written as $\mathbf{D}'_{ij} \cdot (\mathbf{u}_i \times \mathbf{u}_j)$, with $D'_{ij,x} = A'_{ij,yz} = -A'_{ij,zy}$, $D'_{ij,y} = A'_{ij,zx} = -A'_{ij,xz}$, and $D'_{ij,z} = A'_{ij,xy} = -A'_{ij,yx}$ [also termed as the electric Dzyaloshinskii–Moriya interaction, see Equation (6) in the Main Text for details]. Exemplified by the cases of inversion $\bar{1}$ and mirror plane $\mathfrak{m}_x$, the symmetry rules for the $A'_{ij,\alpha\beta}$ components can be derived as follows. The inversion $\bar{1}$ transforms the system (sketched in Supplementary Fig. 9) in such a way: (i) $\bar{1}$ swaps the i_{th} and j_{th} sites (i.e., $i \leftrightarrow j$), and (ii) $\bar{1}$ reverses the dipoles [i.e., $(u_x, u_y, u_z) \to (-u_x, -u_y, -u_z)$]. Overall, the $(u_{i,x}, u_{i,y}, u_{i,z})$ and $(u_{j,x}, u_{j,y}, u_{j,z})$ are transformed, by inversion $\bar{1}$, to $(-u_{j,x}, -u_{j,y}, -u_{j,z})$ and $(-u_{i,x}, -u_{i,y}, -u_{i,z})$, respectively. Consequently, the anti-symmetric exchange interaction [originally written as Supplementary Equation (11)] are transformed to Supplementary Equation (12). System showcasing inversion symmetry $\bar{1}$ implies that $E_{\text{anti-symmetric}} = E'_{\text{anti-symmetric}}$, which implies that $A'_{ij,yz} = 0$, $A'_{ij,zx} = 0$, and $A'_{ij,xy} = 0$ by comparing Supplementary Equation (11) with Supplementary Equation (12). Similarly, the mirror plane $\mathfrak{m}_x$ transforms $(u_{i,x}, u_{i,y}, u_{i,z})$ and $(u_{j,x}, u_{j,y}, u_{j,z})$ to $(-u_{j,x}, u_{j,y}, u_{j,z})$ and $(-u_{i,x}, u_{i,y}, u_{i,z})$, respectively, as shown in Supplementary Table 21. [Note that the mirror plane $\mathfrak{m}_x$ swaps the i_{th} and j_{th} sites and transform the dipole (u_x, u_y, u_z) to $(-u_x, u_y, u_z)$.] Following this, Supplementary Equation (11) is transformed by $\mathfrak{m}_x$ to Supplementary Equation (13). System with $\mathfrak{m}_x$ symmetry indicates $E_{\text{anti-symmetric}} = E''_{\text{anti-symmetric}}$, and the comparison between Supplementary Equation (11) and Supplementary Equation (13) yields $A'_{ij,yz} = 0$. Following such logic, the symmetry rules regarding the $A'_{ij,\alpha\beta}$ components are summarized in Supplementary Table 22. Interestingly, these symmetry rules of the anti-symmetric coupling between electric dipoles coincides with symmetry rules regarding the magnetic Dzyaloshinskii–Moriya interaction (see e.g., Refs. [14, 15]). Indeed, this is governed by the one-to-one correspondence between electric and magnetic Dzyaloshinskii–Moriya interactions, shown in Refs. [13, 16].

$$E_{\text{anti-symmetric}} = A'_{ij,xy}\left(u_{i,x}u_{j,y} - u_{i,y}u_{j,x}\right) + A'_{ij,zx}\left(u_{i,z}u_{j,x} - u_{i,x}u_{j,z}\right) + A'_{ij,yz}\left(u_{i,y}u_{j,z} - u_{i,z}u_{j,y}\right) \quad (11)$$

$$E'_{\text{anti-symmetric}} = A'_{ij,xy}\left(u_{j,x}u_{i,y} - u_{j,y}u_{i,x}\right) + A'_{ij,zx}\left(u_{j,z}u_{i,x} - u_{j,x}u_{i,z}\right) + A'_{ij,yz}\left(u_{j,y}u_{i,z} - u_{j,z}u_{i,y}\right) \quad (12)$$

$$E''_{\text{anti-symmetric}} = A'_{ij,xy}\left(-u_{j,x}u_{i,y} + u_{j,y}u_{i,x}\right) + A'_{ij,zx}\left(-u_{j,z}u_{i,x} + u_{j,x}u_{i,z}\right) + A'_{ij,yz}\left(u_{j,y}u_{i,z} - u_{j,z}u_{i,y}\right) \quad (13)$$

B. The symmetric exchange interaction between electric dipoles

The symmetric exchange interaction between $\mathbf{u}_i$ and $\mathbf{u}_j$ is written as $\sum_{\alpha\beta} S'_{ij,\alpha\beta} u_{i,\alpha} u_{j,\beta}$ (see Supplementary Equation (14) for the expansion), where $\alpha, \beta = x, y, z$ and $S'_{ij,\alpha\beta} = S'_{ij,\beta\alpha}$. We still take the cases of inversion $\bar{1}$ and mirror plane $\mathfrak{m}_x$ as our examples. As shown in Supplementary Table 21 and demonstrated in Supplementary Note 6 (Section A), we show that $\bar{1} : (u_{i,x}, u_{i,y}, u_{i,z}) \to (-u_{j,x}, -u_{j,y}, -u_{j,z}), (u_{j,x}, u_{j,y}, u_{j,z}) \to (-u_{i,x}, -u_{i,y}, -u_{i,z})$ and $\mathfrak{m}_x : (u_{i,x}, u_{i,y}, u_{i,z}) \to (-u_{j,x}, u_{j,y}, u_{j,z}), (u_{j,x}, u_{j,y}, u_{j,z}) \to (-u_{i,x}, u_{i,y}, u_{i,z})$. Hence, Supplementary Equation (14) is transformed by inversion $\bar{1}$ to Supplementary Equation (15). The inversion symmetry of the system implies $E_{\text{symmetric}} = E'_{\text{symmetric}}$. We find that $E_{\text{symmetric}}$ naturally equals $E'_{\text{symmetric}}$ by comparing Supplementary Equation (14) with Supplementary Equation (15). This indicates that the inversion symmetry is compatible with the symmetric exchange interaction, without giving any restrictions on $S'_{ij,\alpha\beta}$ components. Similarly, the mirror plane $\mathfrak{m}_x$ transforms Supplementary Equation (14) to Supplementary Equation (16). The relationship $E_{\text{symmetric}} = E''_{\text{symmetric}}$ implies that $S'_{ij,xy} = S'_{ij,xz} = 0$ and $S'_{ij,yx} = S'_{ij,zx} = 0$ – following that $S'_{ij,\alpha\beta} = S'_{ij,\beta\alpha}$.

$$\begin{aligned} E_{\text{symmetric}} =& S'_{ij,xx}u_{i,x}u_{j,x} + S'_{ij,yy}u_{i,y}u_{j,y} + S'_{ij,z}u_{i,z}u_{j,z} + S'_{ij,xy}(u_{i,x}u_{j,y} + u_{i,y}u_{j,x}) + \\ & S'_{ij,xz}(u_{i,x}u_{j,z} + u_{i,z}u_{j,x}) + S'_{ij,yz}(u_{i,y}u_{j,z} + u_{i,z}u_{j,y}) \end{aligned} \quad (14)$$

$$\begin{aligned} E'_{\text{symmetric}} =& S'_{ij,xx}u_{j,x}u_{i,x} + S'_{ij,yy}u_{j,y}u_{i,y} + S'_{ij,z}u_{j,z}u_{i,z} + S'_{ij,xy}(u_{j,x}u_{i,y} + u_{j,y}u_{i,x}) + \\ & S'_{ij,xz}(u_{j,x}u_{i,z} + u_{j,z}u_{i,x}) + S'_{ij,yz}(u_{j,y}u_{i,z} + u_{j,z}u_{i,y}) \end{aligned} \quad (15)$$

$$E''_{\mathrm{symmetric}} = S'_{ij,xx}u_{j,x}u_{i,x} + S'_{ij,yy}u_{j,y}u_{i,y} + S'_{ij,z}u_{j,z}u_{i,z} - S'_{ij,xy}(u_{j,x}u_{i,y} + u_{j,y}u_{i,x}) - S'_{ij,xz}(u_{j,x}u_{i,z} + u_{j,z}u_{i,x}) + S'_{ij,yz}(u_{j,y}u_{i,z} + u_{j,z}u_{i,y}) \tag{16}$$

Using the similar logic, the symmetry rules regarding the symmetric exchange interaction between electric dipoles are derived and summarized in Supplementary Table 22.

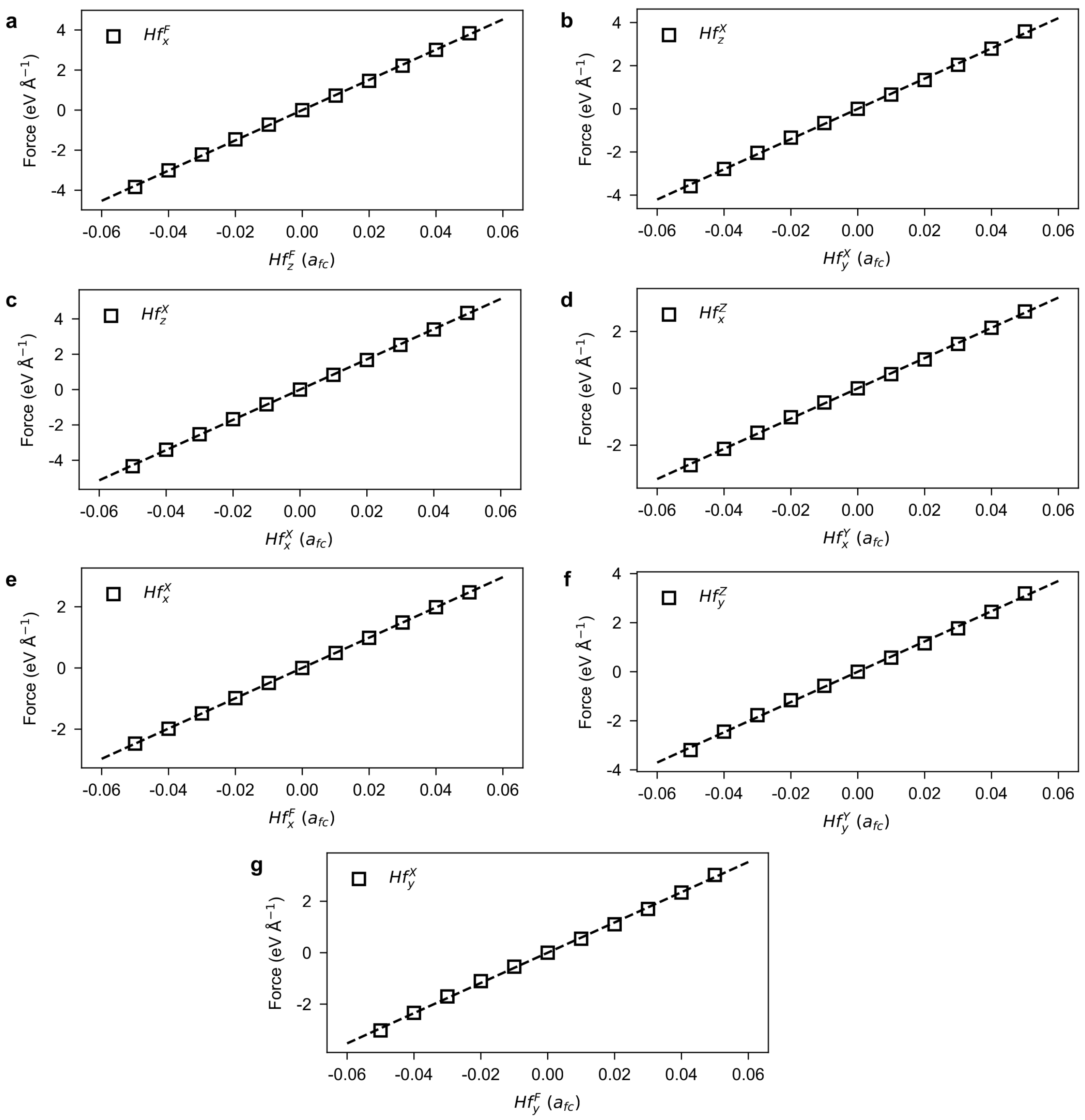

Supplementary Fig. 1. **Numerical verification of various trilinear couplings in H_l ($l = 5 - 11$).** **a** Forces associated with the Hf_x^F mode as a function of Hf_z^F (O_y^G being fixed). **b** Forces associated with the Hf_z^X mode as a function of Hf_y^X (O_x^G being fixed). **c** Forces associated with the Hf_z^X mode as a function of Hf_x^X (O_y^G being fixed). **d** Forces associated with the Hf_x^Z mode as a function of Hf_x^Y ($O_x^{A_x}$ being fixed). **e** Forces associated with the Hf_x^X mode as a function of Hf_x^F ($O_x^{A_x}$ being fixed). **f** Forces associated with the Hf_y^Z mode as a function of Hf_y^Y ($O_x^{A_x}$ being fixed). **g** Forces associated with the Hf_y^X mode as a function of Hf_y^F ($O_x^{A_x}$ being fixed). The dash lines in **a**–**g** display the linear fitting results, corresponding to $Hf_z^F Hf_x^F O_y^G$, $Hf_y^X Hf_z^X O_x^G$, $Hf_x^X Hf_z^X O_y^G$, $Hf_x^Y Hf_x^Z O_x^{A_x}$, $Hf_x^F Hf_x^X O_x^{A_x}$, $Hf_y^Y Hf_y^Z O_x^{A_x}$ and $Hf_y^F Hf_y^X O_x^{A_x}$ couplings, respectively.

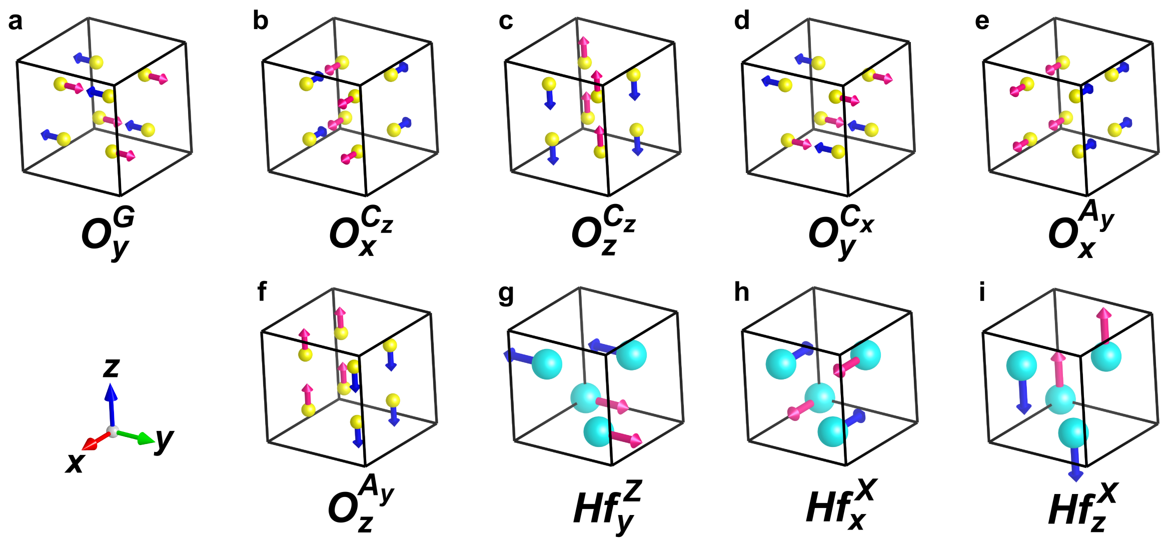

Supplementary Fig. 2. **Structural distortions in the $P2_1/c$ phase of HfO_2 oxide. a-f** Structural distortions associated with the O sublattice. **g-i** Structural distortions associated with the Hf sublattice. The cyan and yellow spheres represent the Hf and O ions, respectively. The blue and pink arrows denote the atomic displacements. The definitions of the notations (e.g., O_y^G and Hf_x^X) are shown in Fig. 1 of the Main Text.

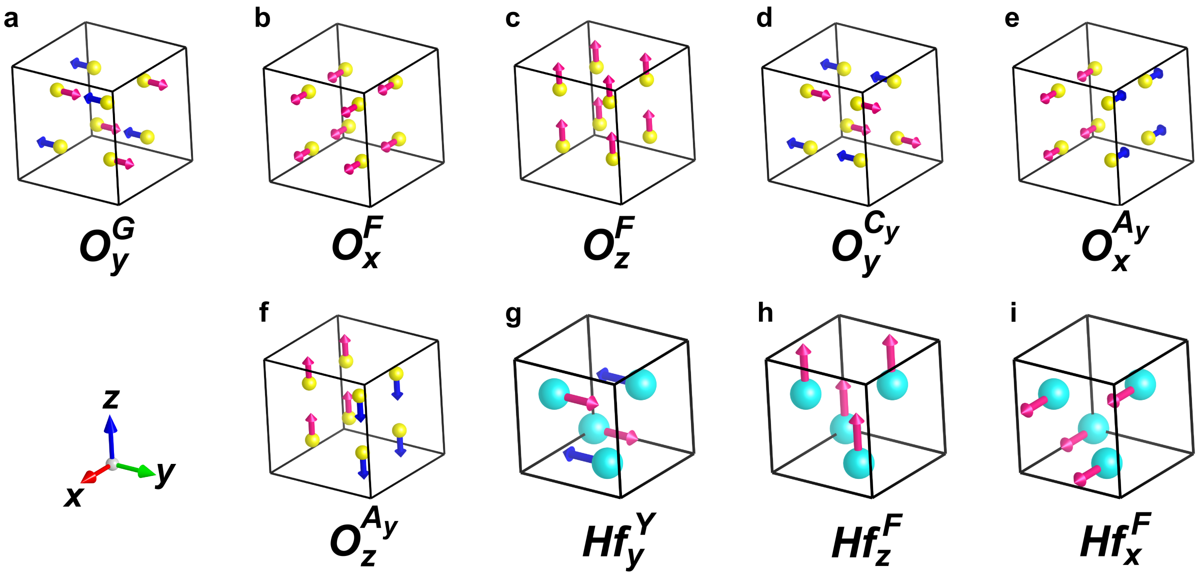

Supplementary Fig. 3. **Structural distortions in the $Pmn2_1$ phase of HfO_2 oxide. a-f** Structural distortions associated with the O sublattice. **g-i** Structural distortions associated with the Hf sublattice. The cyan and yellow spheres represent the Hf and O ions, respectively. The blue and pink arrows denote the atomic displacements. The definitions of the notations (e.g., O_y^G and Hf_y^Y) are shown in Fig. 1 of the Main Text.

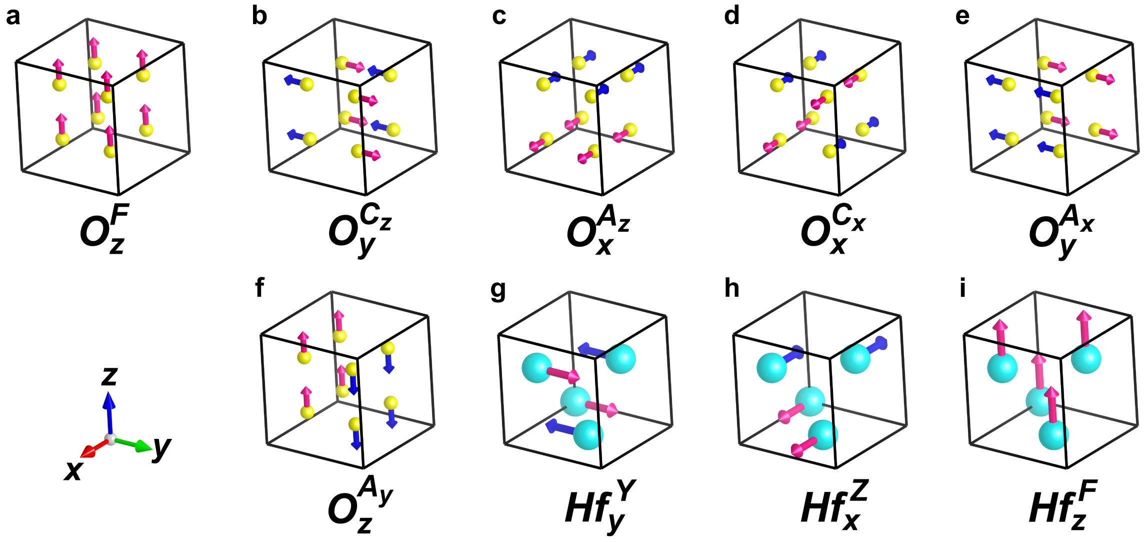

Supplementary Fig. 4. **Structural distortions in the** $Pca2_1$ **phase of** HfO_2 **oxide. a-f** Structural distortions associated with the O sublattice. **g-i** Structural distortions associated with the Hf sublattice. The cyan and yellow spheres represent the Hf and O ions, respectively. The blue and pink arrows denote the atomic displacements. The definitions of the notations (e.g., O_z^F and Hf_y^Y) are shown in Fig. 1 of the Main Text.

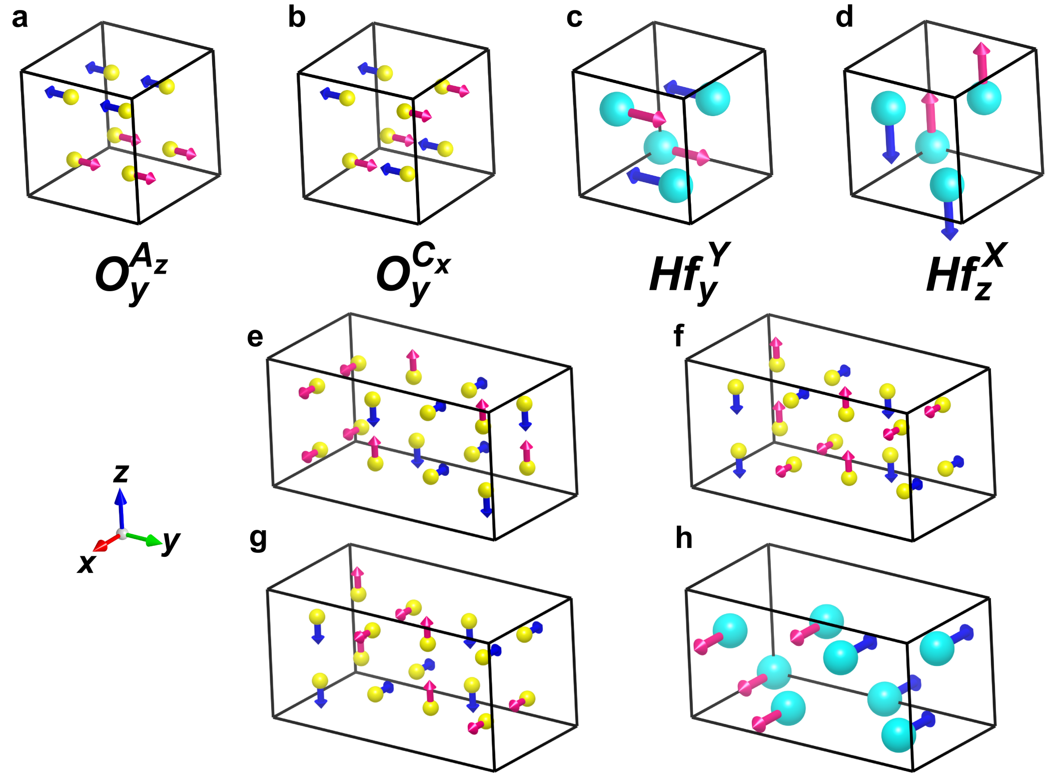

Supplementary Fig. 5. **Structural distortions in the** $Pbca$ **phase of** HfO_2 **oxide. a**, **b**, **e-g** Structural distortions associated with the O sublattice. **c**, **d**, **h** Structural distortions associated with the Hf sublattice. The cyan and yellow spheres represent the Hf and O ions, respectively. The blue and pink arrows denote the atomic displacements. The definitions of notations such as $O_y^{A_z}$ and Hf_z^X shown in panels **a-d** can be found in Fig. 1 of the Main Text. In panels **e-h**, the atomic motions should be defined with respect to a larger cell (compared with the conventional cell of $Fm\bar{3}m$). These configurations can not be labelled according to our convention given in Fig. 1 of the Main Text. Hence, we merely show the atomic motions of these cases without labelling them by any symbols.

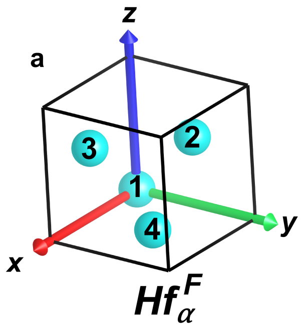

Supplementary Fig. 6. **The lattice modes associated with the Hf sublattice.** **a** The Hf_α^F lattice mode. **b** The Hf_α^X lattice mode. **c** The Hf_α^Y lattice mode. **d** The Hf_α^Z lattice mode. The cyan and pink spheres indicate that the Hf's displacements centered on the corresponding spheres are along $+\alpha$ and $-\alpha$ directions, respectively. For displaying clarity, we do not show the periodic image of the Hf atoms in each cell. Here, each Hf ion is numbered by τ ($\tau = 1, 2, 3, 4$).

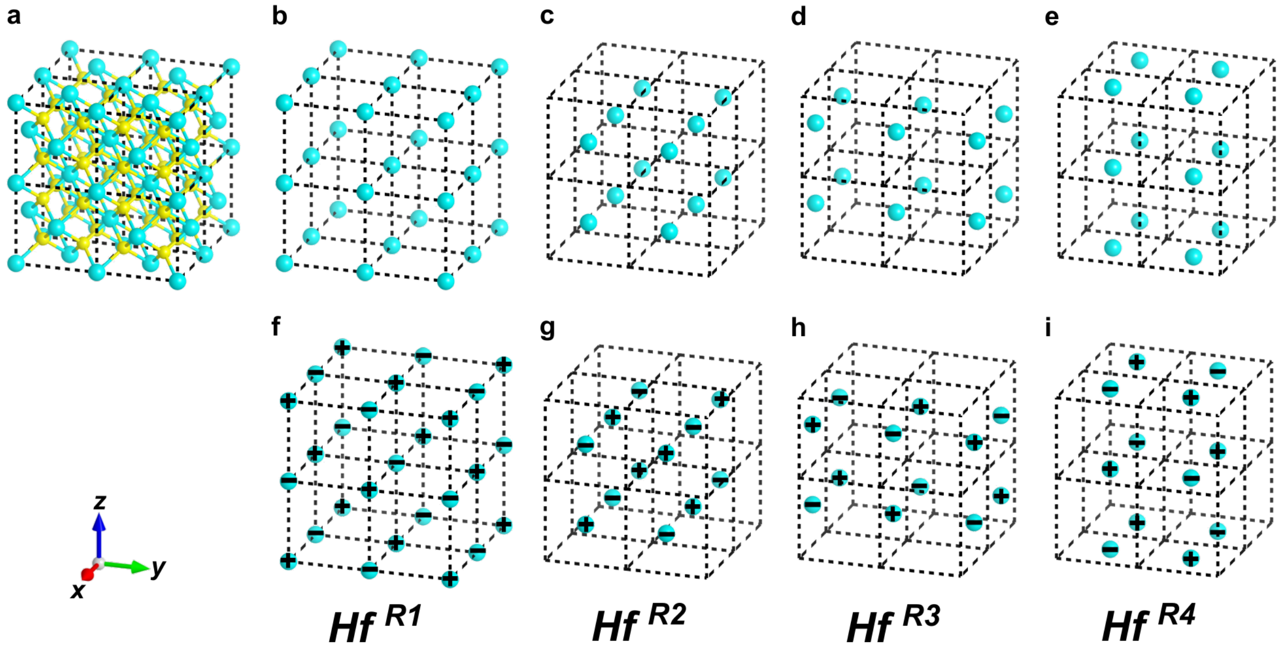

Supplementary Fig. 7. **Several typical order parameters accommodated by the supercell of hafnia.** **a** The $2 \times 2 \times 2$ supercell of the cubic $Fm\bar{3}m$ phase. **b**–**e** Four types of Hf sublattices associated with the cell defined in **a**. **f**–**i** Four lattice modes (labelled as Hf^{R1}, Hf^{R2}, Hf^{R3} and Hf^{R4}) associated with the corresponding Hf sublattices defined in **b**–**e**. Following the procedures sketched in Fig. 1**e** of the Main Text, twelve order parameters [Hf_α^{R1}, Hf_α^{R2}, Hf_α^{R3} and Hf_α^{R4} ($\alpha = x, y, z$)] can be obtained based on the Hf^{R1}, Hf^{R2}, Hf^{R3} and Hf^{R4} lattice modes. In **f**–**i**, the "+" and "−" signs denote that the displacements u_α centered on the Hf sites are along the $+\alpha$ and $-\alpha$ directions, respectively.

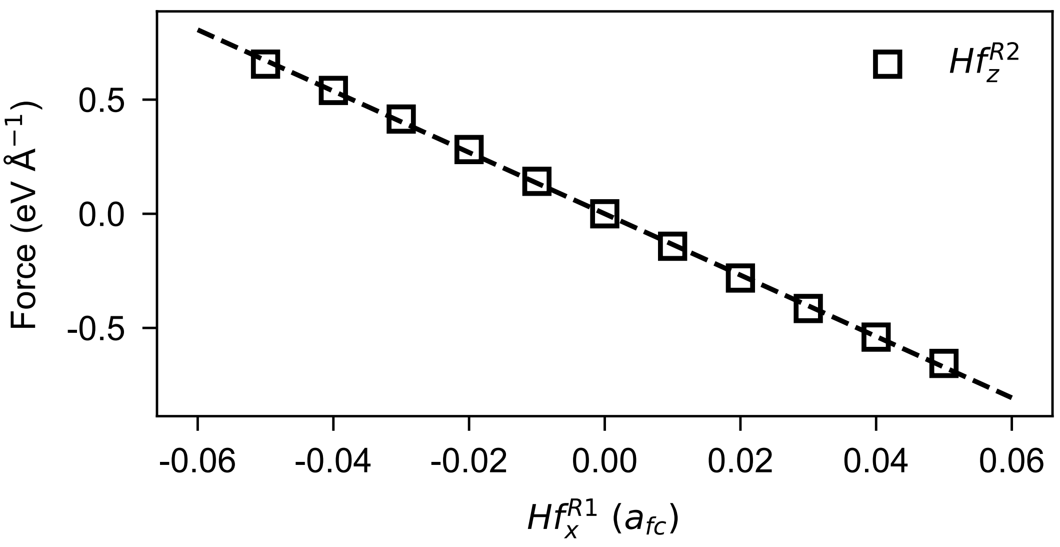

Supplementary Fig. 8. **Numerical verification of the trilinear coupling in H_{sp}.** In this figure, we show forces associated with the Hf_z^{R2} mode as a function of Hf_x^{R1} ($O_x^{A_y}$ being fixed). The dash line denotes the linear fitting result corresponding to $Hf_x^{R1}Hf_z^{R2}O_x^{A_y}$ coupling.

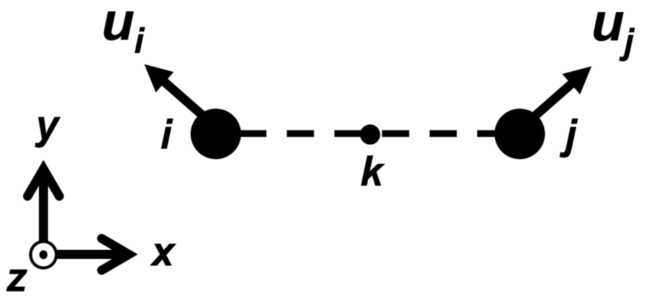

Supplementary Fig. 9. **The coupling between $\mathbf{u}_i$ and $\mathbf{u}_j$ dipoles.** The $\mathbf{u}_i$ and $\mathbf{u}_j$ dipoles reside at the $i_{\rm th}$ and $j_{\rm th}$ sites, respectively. The point k locates in the middle of the $i_{\rm th}$ and $j_{\rm th}$ sites.

Supplementary Table 1. **The transformation rules of various order parameters for HfO_2 under the generators of the $Fm\bar{3}m$ space group.** In this table, the generators 2_{001}, 2_{010} and 2_{110} are two-fold rotations along $\mathbf{z}$, $\mathbf{y}$ and $\mathbf{x}+\mathbf{y}$ directions, respectively; the generator 3^{+}_{111} is the three-fold rotation along the $\mathbf{x}+\mathbf{y}+\mathbf{z}$ direction; $\bar{1}$ is the spatial inversion operation. Furthermore, $t(0,1/2,1/2)$ and $t(1/2,0,1/2)$ denote the $\frac{1}{2}$-lattice-vector translation along $\mathbf{y}+\mathbf{z}$ and $\mathbf{x}+\mathbf{z}$ directions, respectively. In particular, these operations transform the spatial coordinate (x,y,z) as $2_{001}:(x,y,z)\rightarrow(-x,-y,z)$, $2_{010}:(x,y,z)\rightarrow(-x,y,-z)$, $3^{+}_{111}:(x,y,z)\rightarrow(z,x,y)$, $2_{110}:(x,y,z)\rightarrow(y,x,-z)$, $\bar{1}:(x,y,z)\rightarrow(-x,-y,-z)$, $t(0,1/2,1/2):(x,y,z)\rightarrow(x,y+1/2,z+1/2)$, and $t(1/2,0,1/2):(x,y,z)\rightarrow(x+1/2,y,z+1/2)$. Note that our order parameters are defined within the conventional cell of the $Fm\bar{3}m$ of HfO_2. These order parameters are invariant under the $t(1,0,0)$, $t(0,1,0)$, and $t(0,0,1)$ lattice translation operators [e.g., $t(l,m,n):(x,y,z)\rightarrow(x+l,y+m,z+n)$]. Hence, the $t(1,0,0)$, $t(0,1,0)$, and $t(0,0,1)$ generators are not included in our analysis.

	2_{001}	2_{010}	3^{+}_{111}	2_{110}	$\bar{1}$	$t(0,1/2,1/2)$	$t(1/2,0,1/2)$
Hf_x^F	$-Hf_x^F$	$-Hf_x^F$	Hf_y^F	Hf_y^F	$-Hf_x^F$	Hf_x^F	Hf_x^F
Hf_y^F	$-Hf_y^F$	Hf_y^F	Hf_z^F	Hf_x^F	$-Hf_y^F$	Hf_y^F	Hf_y^F
Hf_z^F	Hf_z^F	$-Hf_z^F$	Hf_x^F	$-Hf_z^F$	$-Hf_z^F$	Hf_z^F	Hf_z^F
Hf_x^X	$-Hf_x^X$	$-Hf_x^X$	Hf_y^Y	Hf_y^Y	$-Hf_x^X$	Hf_x^X	$-Hf_x^X$
Hf_y^Y	$-Hf_y^Y$	Hf_y^Y	Hf_z^Z	Hf_x^X	$-Hf_y^Y$	$-Hf_y^Y$	Hf_y^Y
Hf_z^Z	Hf_z^Z	$-Hf_z^Z$	Hf_x^X	$-Hf_z^Z$	$-Hf_z^Z$	$-Hf_z^Z$	$-Hf_z^Z$
Hf_y^X	$-Hf_y^X$	Hf_y^X	Hf_z^Y	Hf_x^Y	$-Hf_y^X$	Hf_y^X	$-Hf_y^X$
Hf_z^X	Hf_z^X	$-Hf_z^X$	Hf_x^Y	$-Hf_z^Y$	$-Hf_z^X$	Hf_z^X	$-Hf_z^X$
Hf_x^Y	$-Hf_x^Y$	$-Hf_x^Y$	Hf_y^Z	Hf_y^X	$-Hf_x^Y$	$-Hf_x^Y$	Hf_x^Y
Hf_z^Y	Hf_z^Y	$-Hf_z^Y$	Hf_x^Z	$-Hf_z^X$	$-Hf_z^Y$	$-Hf_z^Y$	Hf_z^Y
Hf_x^Z	$-Hf_x^Z$	$-Hf_x^Z$	Hf_y^X	Hf_y^Z	$-Hf_x^Z$	$-Hf_x^Z$	$-Hf_x^Z$
Hf_y^Z	$-Hf_y^Z$	Hf_y^Z	Hf_z^X	Hf_x^Z	$-Hf_y^Z$	$-Hf_y^Z$	$-Hf_y^Z$
O_x^F	$-O_x^F$	$-O_x^F$	O_y^F	O_y^F	$-O_x^F$	O_x^F	O_x^F
O_y^F	$-O_y^F$	O_y^F	O_z^F	O_x^F	$-O_y^F$	O_y^F	O_y^F
O_z^F	O_z^F	$-O_z^F$	O_x^F	$-O_z^F$	$-O_z^F$	O_z^F	O_z^F
O_x^G	$-O_x^G$	$-O_x^G$	O_y^G	$-O_y^G$	O_x^G	O_x^G	O_x^G
O_y^G	$-O_y^G$	O_y^G	O_z^G	$-O_x^G$	O_y^G	O_y^G	O_y^G
O_z^G	O_z^G	$-O_z^G$	O_x^G	O_z^G	O_z^G	O_z^G	O_z^G
$O_x^{A_x}$	$O_x^{A_x}$	$O_x^{A_x}$	$O_y^{A_y}$	$O_y^{A_y}$	$O_x^{A_x}$	$O_x^{A_x}$	$-O_x^{A_x}$
$O_y^{A_y}$	$O_y^{A_y}$	$O_y^{A_y}$	$O_z^{A_z}$	$O_x^{A_x}$	$O_y^{A_y}$	$-O_y^{A_y}$	$O_y^{A_y}$
$O_z^{A_z}$	$O_z^{A_z}$	$O_z^{A_z}$	$O_x^{A_x}$	$O_z^{A_z}$	$O_z^{A_z}$	$-O_z^{A_z}$	$-O_z^{A_z}$
$O_y^{A_x}$	$O_y^{A_x}$	$-O_y^{A_x}$	$O_z^{A_y}$	$O_x^{A_y}$	$O_y^{A_x}$	$O_y^{A_x}$	$-O_y^{A_x}$
$O_z^{A_x}$	$-O_z^{A_x}$	$O_z^{A_x}$	$O_x^{A_y}$	$-O_z^{A_y}$	$O_z^{A_x}$	$O_z^{A_x}$	$-O_z^{A_x}$
$O_x^{A_y}$	$O_x^{A_y}$	$-O_x^{A_y}$	$O_y^{A_z}$	$O_y^{A_x}$	$O_x^{A_y}$	$-O_x^{A_y}$	$O_x^{A_y}$
$O_z^{A_y}$	$-O_z^{A_y}$	$-O_z^{A_y}$	$O_x^{A_z}$	$-O_z^{A_x}$	$O_z^{A_y}$	$-O_z^{A_y}$	$O_z^{A_y}$
$O_x^{A_z}$	$-O_x^{A_z}$	$O_x^{A_z}$	$O_y^{A_x}$	$-O_y^{A_z}$	$O_x^{A_z}$	$-O_x^{A_z}$	$-O_x^{A_z}$
$O_y^{A_z}$	$-O_y^{A_z}$	$-O_y^{A_z}$	$O_z^{A_x}$	$-O_x^{A_z}$	$O_y^{A_z}$	$-O_y^{A_z}$	$-O_y^{A_z}$
$O_x^{C_x}$	$O_x^{C_x}$	$O_x^{C_x}$	$O_y^{C_y}$	$-O_y^{C_y}$	$-O_x^{C_x}$	$O_x^{C_x}$	$-O_x^{C_x}$
$O_y^{C_y}$	$O_y^{C_y}$	$O_y^{C_y}$	$O_z^{C_z}$	$-O_x^{C_x}$	$-O_y^{C_y}$	$-O_y^{C_y}$	$O_y^{C_y}$
$O_z^{C_z}$	$O_z^{C_z}$	$O_z^{C_z}$	$O_x^{C_x}$	$-O_z^{C_z}$	$-O_z^{C_z}$	$-O_z^{C_z}$	$-O_z^{C_z}$
$O_y^{C_x}$	$O_y^{C_x}$	$-O_y^{C_x}$	$O_z^{C_y}$	$-O_x^{C_y}$	$-O_y^{C_x}$	$O_y^{C_x}$	$-O_y^{C_x}$
$O_z^{C_x}$	$-O_z^{C_x}$	$O_z^{C_x}$	$O_x^{C_y}$	$O_z^{C_y}$	$-O_z^{C_x}$	$O_z^{C_x}$	$-O_z^{C_x}$
$O_x^{C_y}$	$O_x^{C_y}$	$-O_x^{C_y}$	$O_y^{C_z}$	$-O_y^{C_x}$	$-O_x^{C_y}$	$-O_x^{C_y}$	$O_x^{C_y}$
$O_z^{C_y}$	$-O_z^{C_y}$	$-O_z^{C_y}$	$O_x^{C_z}$	$O_z^{C_x}$	$-O_z^{C_y}$	$-O_z^{C_y}$	$O_z^{C_y}$
$O_x^{C_z}$	$-O_x^{C_z}$	$O_x^{C_z}$	$O_y^{C_x}$	$O_y^{C_z}$	$-O_x^{C_z}$	$-O_x^{C_z}$	$-O_x^{C_z}$
$O_y^{C_z}$	$-O_y^{C_z}$	$-O_y^{C_z}$	$O_z^{C_x}$	$O_x^{C_z}$	$-O_y^{C_z}$	$-O_y^{C_z}$	$-O_y^{C_z}$

Supplementary Table 2. **The anti-symmetric exchange interaction $A'_{\tau\kappa}$ between the dipoles centered on $\mathbf{R}_m + \mathbf{r}_\tau$ and $\mathbf{R}_m + \mathbf{r}_\kappa$.** The tensor $A'_{\tau\kappa}$ is derived with respect to H_1.

$\mathbf{r}_\kappa$ / $\mathbf{r}_\tau$	$(0,0,0)$	$(0,\frac{1}{2},\frac{1}{2})$
$(0,0,0)$	N/A	$\begin{pmatrix} 0 & -O_x^{A_y} & -O_x^{A_z} \\ O_x^{A_y} & 0 & O_y^{A_z} - O_z^{A_y} \\ O_x^{A_z} & O_z^{A_y} - O_y^{A_z} & 0 \end{pmatrix}$
$(0,\frac{1}{2},\frac{1}{2})$	$\begin{pmatrix} 0 & O_x^{A_y} & O_x^{A_z} \\ -O_x^{A_y} & 0 & O_z^{A_y} - O_y^{A_z} \\ -O_x^{A_z} & O_y^{A_z} - O_z^{A_y} & 0 \end{pmatrix}$	N/A
$(\frac{1}{2},0,\frac{1}{2})$	$\begin{pmatrix} 0 & -O_y^{A_x} & O_z^{A_x} - O_x^{A_z} \\ O_y^{A_x} & 0 & O_y^{A_z} \\ O_x^{A_z} - O_z^{A_x} & -O_y^{A_z} & 0 \end{pmatrix}$	$\begin{pmatrix} 0 & O_y^{A_x} + O_x^{A_y} & -O_z^{A_x} \\ -O_y^{A_x} - O_x^{A_y} & 0 & O_z^{A_y} \\ O_z^{A_x} & -O_z^{A_y} & 0 \end{pmatrix}$
$(\frac{1}{2},\frac{1}{2},0)$	$\begin{pmatrix} 0 & O_y^{A_x} - O_x^{A_y} & -O_z^{A_x} \\ O_x^{A_y} - O_y^{A_x} & 0 & -O_z^{A_y} \\ O_z^{A_x} & O_z^{A_y} & 0 \end{pmatrix}$	$\begin{pmatrix} 0 & -O_y^{A_x} & O_z^{A_x} + O_x^{A_z} \\ O_y^{A_x} & 0 & -O_y^{A_z} \\ -O_z^{A_x} - O_x^{A_z} & O_y^{A_z} & 0 \end{pmatrix}$

$\mathbf{r}_\kappa$ / $\mathbf{r}_\tau$	$(\frac{1}{2},0,\frac{1}{2})$	$(\frac{1}{2},\frac{1}{2},0)$
$(0,0,0)$	$\begin{pmatrix} 0 & O_y^{A_x} & O_x^{A_z} - O_z^{A_x} \\ -O_y^{A_x} & 0 & -O_y^{A_z} \\ O_z^{A_x} - O_x^{A_z} & O_y^{A_z} & 0 \end{pmatrix}$	$\begin{pmatrix} 0 & O_x^{A_y} - O_y^{A_x} & O_z^{A_x} \\ O_y^{A_x} - O_x^{A_y} & 0 & O_z^{A_y} \\ -O_z^{A_x} & -O_z^{A_y} & 0 \end{pmatrix}$
$(0,\frac{1}{2},\frac{1}{2})$	$\begin{pmatrix} 0 & -O_y^{A_x} - O_x^{A_y} & O_z^{A_x} \\ O_y^{A_x} + O_x^{A_y} & 0 & -O_z^{A_y} \\ -O_z^{A_x} & O_z^{A_y} & 0 \end{pmatrix}$	$\begin{pmatrix} 0 & O_y^{A_x} & -O_z^{A_x} - O_x^{A_z} \\ -O_y^{A_x} & 0 & O_y^{A_z} \\ O_z^{A_x} + O_x^{A_z} & -O_y^{A_z} & 0 \end{pmatrix}$
$(\frac{1}{2},0,\frac{1}{2})$	N/A	$\begin{pmatrix} 0 & -O_x^{A_y} & O_x^{A_z} \\ O_x^{A_y} & 0 & -O_z^{A_y} - O_y^{A_z} \\ -O_x^{A_z} & O_z^{A_y} + O_y^{A_z} & 0 \end{pmatrix}$
$(\frac{1}{2},\frac{1}{2},0)$	$\begin{pmatrix} 0 & O_x^{A_y} & -O_x^{A_z} \\ -O_x^{A_y} & 0 & O_z^{A_y} + O_y^{A_z} \\ O_x^{A_z} & -O_z^{A_y} - O_y^{A_z} & 0 \end{pmatrix}$	N/A

Supplementary Table 3. **The anisotropic symmetric exchange interaction $S'_{\tau\kappa}$ between the dipoles centered on $\mathbf{R}_m + \mathbf{r}_\tau$ and $\mathbf{R}_m + \mathbf{r}_\kappa$.** The tensor $S'_{\tau\kappa}$ is derived with respect to H_1.

$\mathbf{r}_\tau$ \ $\mathbf{r}_\kappa$	$(0,0,0)$	$(0,\frac{1}{2},\frac{1}{2})$
$(0,0,0)$	N/A	$\begin{pmatrix} 0 & -O_y^{A_x} & -O_z^{A_x} \\ -O_y^{A_x} & 0 & 0 \\ -O_z^{A_x} & 0 & 0 \end{pmatrix}$
$(0,\frac{1}{2},\frac{1}{2})$	$\begin{pmatrix} 0 & -O_y^{A_x} & -O_z^{A_x} \\ -O_y^{A_x} & 0 & 0 \\ -O_z^{A_x} & 0 & 0 \end{pmatrix}$	N/A
$(\frac{1}{2},0,\frac{1}{2})$	$\begin{pmatrix} 0 & -O_x^{A_y} & 0 \\ -O_x^{A_y} & 0 & -O_z^{A_y} \\ 0 & -O_z^{A_y} & 0 \end{pmatrix}$	$\begin{pmatrix} 0 & 0 & O_x^{A_z} \\ 0 & 0 & O_y^{A_z} \\ O_x^{A_z} & O_y^{A_z} & 0 \end{pmatrix}$
$(\frac{1}{2},\frac{1}{2},0)$	$\begin{pmatrix} 0 & 0 & -O_x^{A_z} \\ 0 & 0 & -O_y^{A_z} \\ -O_x^{A_z} & -O_y^{A_z} & 0 \end{pmatrix}$	$\begin{pmatrix} 0 & O_x^{A_y} & 0 \\ O_x^{A_y} & 0 & O_z^{A_y} \\ 0 & O_z^{A_y} & 0 \end{pmatrix}$

$\mathbf{r}_\tau$ \ $\mathbf{r}_\kappa$	$(\frac{1}{2},0,\frac{1}{2})$	$(\frac{1}{2},\frac{1}{2},0)$
$(0,0,0)$	$\begin{pmatrix} 0 & -O_x^{A_y} & 0 \\ -O_x^{A_y} & 0 & -O_z^{A_y} \\ 0 & -O_z^{A_y} & 0 \end{pmatrix}$	$\begin{pmatrix} 0 & 0 & -O_x^{A_z} \\ 0 & 0 & -O_y^{A_z} \\ -O_x^{A_z} & -O_y^{A_z} & 0 \end{pmatrix}$
$(0,\frac{1}{2},\frac{1}{2})$	$\begin{pmatrix} 0 & 0 & O_x^{A_z} \\ 0 & 0 & O_y^{A_z} \\ O_x^{A_z} & O_y^{A_z} & 0 \end{pmatrix}$	$\begin{pmatrix} 0 & O_x^{A_y} & 0 \\ O_x^{A_y} & 0 & O_z^{A_y} \\ 0 & O_z^{A_y} & 0 \end{pmatrix}$
$(\frac{1}{2},0,\frac{1}{2})$	N/A	$\begin{pmatrix} 0 & O_y^{A_x} & O_z^{A_x} \\ O_y^{A_x} & 0 & 0 \\ O_z^{A_x} & 0 & 0 \end{pmatrix}$
$(\frac{1}{2},\frac{1}{2},0)$	$\begin{pmatrix} 0 & O_y^{A_x} & O_z^{A_x} \\ O_y^{A_x} & 0 & 0 \\ O_z^{A_x} & 0 & 0 \end{pmatrix}$	N/A

Supplementary Table 4. **The anti-symmetric exchange interaction $A'_{\tau\kappa}$ between the dipoles centered on $\mathbf{R}_m + \mathbf{r}_\tau$ and $\mathbf{R}_m + \mathbf{r}_\kappa$.** The tensor $A'_{\tau\kappa}$ is derived with respect to H_2.

$\mathbf{r}_\tau$ \ $\mathbf{r}_\kappa$	$(0,0,0)$	$(0,\frac{1}{2},\frac{1}{2})$
$(0,0,0)$	N/A	$\begin{pmatrix} 0 & -O_x^{A_y} & -O_x^{A_z} \\ O_x^{A_y} & 0 & O_z^{A_y} - O_y^{A_z} \\ O_x^{A_z} & O_y^{A_z} - O_z^{A_y} & 0 \end{pmatrix}$
$(0,\frac{1}{2},\frac{1}{2})$	$\begin{pmatrix} 0 & O_x^{A_y} & O_x^{A_z} \\ -O_x^{A_y} & 0 & O_y^{A_z} - O_z^{A_y} \\ -O_x^{A_z} & O_z^{A_y} - O_y^{A_z} & 0 \end{pmatrix}$	N/A
$(\frac{1}{2},0,\frac{1}{2})$	$\begin{pmatrix} 0 & -O_y^{A_x} & O_x^{A_z} - O_z^{A_x} \\ O_y^{A_x} & 0 & O_y^{A_z} \\ O_z^{A_x} - O_x^{A_z} & -O_y^{A_z} & 0 \end{pmatrix}$	$\begin{pmatrix} 0 & -O_y^{A_x} - O_x^{A_y} & -O_z^{A_x} \\ O_y^{A_x} + O_x^{A_y} & 0 & O_z^{A_y} \\ O_z^{A_x} & -O_z^{A_y} & 0 \end{pmatrix}$
$(\frac{1}{2},\frac{1}{2},0)$	$\begin{pmatrix} 0 & O_x^{A_y} - O_y^{A_x} & -O_z^{A_x} \\ O_y^{A_x} - O_x^{A_y} & 0 & -O_z^{A_y} \\ O_z^{A_x} & O_z^{A_y} & 0 \end{pmatrix}$	$\begin{pmatrix} 0 & -O_y^{A_x} & -O_z^{A_x} - O_x^{A_z} \\ O_y^{A_x} & 0 & -O_y^{A_z} \\ O_z^{A_x} + O_x^{A_z} & O_y^{A_z} & 0 \end{pmatrix}$

$\mathbf{r}_\tau$ \ $\mathbf{r}_\kappa$	$(\frac{1}{2},0,\frac{1}{2})$	$(\frac{1}{2},\frac{1}{2},0)$
$(0,0,0)$	$\begin{pmatrix} 0 & O_y^{A_x} & O_z^{A_x} - O_x^{A_z} \\ -O_y^{A_x} & 0 & -O_y^{A_z} \\ O_x^{A_z} - O_z^{A_x} & O_y^{A_z} & 0 \end{pmatrix}$	$\begin{pmatrix} 0 & O_y^{A_x} - O_x^{A_y} & O_z^{A_x} \\ O_x^{A_y} - O_y^{A_x} & 0 & O_z^{A_y} \\ -O_z^{A_x} & -O_z^{A_y} & 0 \end{pmatrix}$
$(0,\frac{1}{2},\frac{1}{2})$	$\begin{pmatrix} 0 & O_y^{A_x} + O_x^{A_y} & O_z^{A_x} \\ -O_y^{A_x} - O_x^{A_y} & 0 & -O_z^{A_y} \\ -O_z^{A_x} & O_z^{A_y} & 0 \end{pmatrix}$	$\begin{pmatrix} 0 & O_y^{A_x} & O_z^{A_x} + O_x^{A_z} \\ -O_y^{A_x} & 0 & O_y^{A_z} \\ -O_z^{A_x} - O_x^{A_z} & -O_y^{A_z} & 0 \end{pmatrix}$
$(\frac{1}{2},0,\frac{1}{2})$	N/A	$\begin{pmatrix} 0 & -O_x^{A_y} & O_x^{A_z} \\ O_x^{A_y} & 0 & O_z^{A_y} + O_y^{A_z} \\ -O_x^{A_z} & -O_z^{A_y} - O_y^{A_z} & 0 \end{pmatrix}$
$(\frac{1}{2},\frac{1}{2},0)$	$\begin{pmatrix} 0 & O_x^{A_y} & -O_x^{A_z} \\ -O_x^{A_y} & 0 & -O_z^{A_y} - O_y^{A_z} \\ O_x^{A_z} & O_z^{A_y} + O_y^{A_z} & 0 \end{pmatrix}$	N/A

Supplementary Table 5. **The anisotropic symmetric exchange interaction $S'_{\tau\kappa}$ between the dipoles centered on $\mathbf{R}_m + \mathbf{r}_\tau$ and $\mathbf{R}_m + \mathbf{r}_\kappa$.** The tensor $S'_{\tau\kappa}$ is derived with respect to H_2.

$\mathbf{r}_\tau$ \ $\mathbf{r}_\kappa$	$(0,0,0)$	$(0,\frac{1}{2},\frac{1}{2})$
$(0,0,0)$	N/A	$\begin{pmatrix} 0 & O_y^{A_x} & O_z^{A_x} \\ O_y^{A_x} & 0 & 0 \\ O_z^{A_x} & 0 & 0 \end{pmatrix}$
$(0,\frac{1}{2},\frac{1}{2})$	$\begin{pmatrix} 0 & O_y^{A_x} & O_z^{A_x} \\ O_y^{A_x} & 0 & 0 \\ O_z^{A_x} & 0 & 0 \end{pmatrix}$	N/A
$(\frac{1}{2},0,\frac{1}{2})$	$\begin{pmatrix} 0 & O_x^{A_y} & 0 \\ O_x^{A_y} & 0 & O_z^{A_y} \\ 0 & O_z^{A_y} & 0 \end{pmatrix}$	$\begin{pmatrix} 0 & 0 & -O_x^{A_z} \\ 0 & 0 & -O_y^{A_z} \\ -O_x^{A_z} & -O_y^{A_z} & 0 \end{pmatrix}$
$(\frac{1}{2},\frac{1}{2},0)$	$\begin{pmatrix} 0 & 0 & O_x^{A_z} \\ 0 & 0 & O_y^{A_z} \\ O_x^{A_z} & O_y^{A_z} & 0 \end{pmatrix}$	$\begin{pmatrix} 0 & -O_x^{A_y} & 0 \\ -O_x^{A_y} & 0 & -O_z^{A_y} \\ 0 & -O_z^{A_y} & 0 \end{pmatrix}$

$\mathbf{r}_\tau$ \ $\mathbf{r}_\kappa$	$(\frac{1}{2},0,\frac{1}{2})$	$(\frac{1}{2},\frac{1}{2},0)$
$(0,0,0)$	$\begin{pmatrix} 0 & O_x^{A_y} & 0 \\ O_x^{A_y} & 0 & O_z^{A_y} \\ 0 & O_z^{A_y} & 0 \end{pmatrix}$	$\begin{pmatrix} 0 & 0 & O_x^{A_z} \\ 0 & 0 & O_y^{A_z} \\ O_x^{A_z} & O_y^{A_z} & 0 \end{pmatrix}$
$(0,\frac{1}{2},\frac{1}{2})$	$\begin{pmatrix} 0 & 0 & -O_x^{A_z} \\ 0 & 0 & -O_y^{A_z} \\ -O_x^{A_z} & -O_y^{A_z} & 0 \end{pmatrix}$	$\begin{pmatrix} 0 & -O_x^{A_y} & 0 \\ -O_x^{A_y} & 0 & -O_z^{A_y} \\ 0 & -O_z^{A_y} & 0 \end{pmatrix}$
$(\frac{1}{2},0,\frac{1}{2})$	N/A	$\begin{pmatrix} 0 & -O_y^{A_x} & -O_z^{A_x} \\ -O_y^{A_x} & 0 & 0 \\ -O_z^{A_x} & 0 & 0 \end{pmatrix}$
$(\frac{1}{2},\frac{1}{2},0)$	$\begin{pmatrix} 0 & -O_y^{A_x} & -O_z^{A_x} \\ -O_y^{A_x} & 0 & 0 \\ -O_z^{A_x} & 0 & 0 \end{pmatrix}$	N/A

Supplementary Table 6. **The anti-symmetric exchange interaction $A'_{\tau\kappa}$ between the dipoles centered on $\mathbf{R}_m + \mathbf{r}_\tau$ and $\mathbf{R}_m + \mathbf{r}_\kappa$.** The tensor $A'_{\tau\kappa}$ is derived with respect to H_3.

$\mathbf{r}_\tau$ \ $\mathbf{r}_\kappa$	$(0,0,0)$	$(0,\frac{1}{2},\frac{1}{2})$
$(0,0,0)$	N/A	$\begin{pmatrix} 0 & O_x^{A_y} & O_x^{A_z} \\ -O_x^{A_y} & 0 & O_y^{A_z} - O_z^{A_y} \\ -O_x^{A_z} & O_z^{A_y} - O_y^{A_z} & 0 \end{pmatrix}$
$(0,\frac{1}{2},\frac{1}{2})$	$\begin{pmatrix} 0 & -O_x^{A_y} & -O_x^{A_z} \\ O_x^{A_y} & 0 & O_z^{A_y} - O_y^{A_z} \\ O_x^{A_z} & O_y^{A_z} - O_z^{A_y} & 0 \end{pmatrix}$	N/A
$(\frac{1}{2},0,\frac{1}{2})$	$\begin{pmatrix} 0 & O_y^{A_x} & O_z^{A_x} - O_x^{A_z} \\ -O_y^{A_x} & 0 & -O_y^{A_z} \\ O_x^{A_z} - O_z^{A_x} & O_y^{A_z} & 0 \end{pmatrix}$	$\begin{pmatrix} 0 & O_y^{A_x} + O_x^{A_y} & O_z^{A_x} \\ -O_y^{A_x} - O_x^{A_y} & 0 & -O_z^{A_y} \\ -O_z^{A_x} & O_z^{A_y} & 0 \end{pmatrix}$
$(\frac{1}{2},\frac{1}{2},0)$	$\begin{pmatrix} 0 & O_y^{A_x} - O_x^{A_y} & O_z^{A_x} \\ O_x^{A_y} - O_y^{A_x} & 0 & O_z^{A_y} \\ -O_z^{A_x} & -O_z^{A_y} & 0 \end{pmatrix}$	$\begin{pmatrix} 0 & O_y^{A_x} & O_z^{A_x} + O_x^{A_z} \\ -O_y^{A_x} & 0 & O_y^{A_z} \\ -O_z^{A_x} - O_x^{A_z} & -O_y^{A_z} & 0 \end{pmatrix}$

$\mathbf{r}_\tau$ \ $\mathbf{r}_\kappa$	$(\frac{1}{2},0,\frac{1}{2})$	$(\frac{1}{2},\frac{1}{2},0)$
$(0,0,0)$	$\begin{pmatrix} 0 & -O_y^{A_x} & O_x^{A_z} - O_z^{A_x} \\ O_y^{A_x} & 0 & O_y^{A_z} \\ O_z^{A_x} - O_x^{A_z} & -O_y^{A_z} & 0 \end{pmatrix}$	$\begin{pmatrix} 0 & O_x^{A_y} - O_y^{A_x} & -O_z^{A_x} \\ O_y^{A_x} - O_x^{A_y} & 0 & -O_z^{A_y} \\ O_z^{A_x} & O_z^{A_y} & 0 \end{pmatrix}$
$(0,\frac{1}{2},\frac{1}{2})$	$\begin{pmatrix} 0 & -O_y^{A_x} - O_x^{A_y} & -O_z^{A_x} \\ O_y^{A_x} + O_x^{A_y} & 0 & O_z^{A_y} \\ O_z^{A_x} & -O_z^{A_y} & 0 \end{pmatrix}$	$\begin{pmatrix} 0 & -O_y^{A_x} & -O_z^{A_x} - O_x^{A_z} \\ O_y^{A_x} & 0 & -O_y^{A_z} \\ O_z^{A_x} + O_x^{A_z} & O_y^{A_z} & 0 \end{pmatrix}$
$(\frac{1}{2},0,\frac{1}{2})$	N/A	$\begin{pmatrix} 0 & O_x^{A_y} & -O_x^{A_z} \\ -O_x^{A_y} & 0 & -O_z^{A_y} - O_y^{A_z} \\ O_x^{A_z} & O_z^{A_y} + O_y^{A_z} & 0 \end{pmatrix}$
$(\frac{1}{2},\frac{1}{2},0)$	$\begin{pmatrix} 0 & -O_x^{A_y} & O_x^{A_z} \\ O_x^{A_y} & 0 & O_z^{A_y} + O_y^{A_z} \\ -O_x^{A_z} & -O_z^{A_y} - O_y^{A_z} & 0 \end{pmatrix}$	N/A

Supplementary Table 7. **The anisotropic symmetric exchange interaction $S'_{\tau\kappa}$ between the dipoles centered on $\mathbf{R}_m + \mathbf{r}_\tau$ and $\mathbf{R}_m + \mathbf{r}_\kappa$.** The tensor $S'_{\tau\kappa}$ is derived with respect to H_3.

$\mathbf{r}_\tau$ \ $\mathbf{r}_\kappa$	$(0,0,0)$	$(0,\frac{1}{2},\frac{1}{2})$
$(0,0,0)$	N/A	$\begin{pmatrix} 0 & O_y^{A_x} & O_z^{A_x} \\ O_y^{A_x} & 0 & 0 \\ O_z^{A_x} & 0 & 0 \end{pmatrix}$
$(0,\frac{1}{2},\frac{1}{2})$	$\begin{pmatrix} 0 & O_y^{A_x} & O_z^{A_x} \\ O_y^{A_x} & 0 & 0 \\ O_z^{A_x} & 0 & 0 \end{pmatrix}$	N/A
$(\frac{1}{2},0,\frac{1}{2})$	$\begin{pmatrix} 0 & O_x^{A_y} & 0 \\ O_x^{A_y} & 0 & O_z^{A_y} \\ 0 & O_z^{A_y} & 0 \end{pmatrix}$	$\begin{pmatrix} 0 & 0 & -O_x^{A_z} \\ 0 & 0 & -O_y^{A_z} \\ -O_x^{A_z} & -O_y^{A_z} & 0 \end{pmatrix}$
$(\frac{1}{2},\frac{1}{2},0)$	$\begin{pmatrix} 0 & 0 & O_x^{A_z} \\ 0 & 0 & O_y^{A_z} \\ O_x^{A_z} & O_y^{A_z} & 0 \end{pmatrix}$	$\begin{pmatrix} 0 & -O_x^{A_y} & 0 \\ -O_x^{A_y} & 0 & -O_z^{A_y} \\ 0 & -O_z^{A_y} & 0 \end{pmatrix}$

$\mathbf{r}_\tau$ \ $\mathbf{r}_\kappa$	$(\frac{1}{2},0,\frac{1}{2})$	$(\frac{1}{2},\frac{1}{2},0)$
$(0,0,0)$	$\begin{pmatrix} 0 & O_x^{A_y} & 0 \\ O_x^{A_y} & 0 & O_z^{A_y} \\ 0 & O_z^{A_y} & 0 \end{pmatrix}$	$\begin{pmatrix} 0 & 0 & O_x^{A_z} \\ 0 & 0 & O_y^{A_z} \\ O_x^{A_z} & O_y^{A_z} & 0 \end{pmatrix}$
$(0,\frac{1}{2},\frac{1}{2})$	$\begin{pmatrix} 0 & 0 & -O_x^{A_z} \\ 0 & 0 & -O_y^{A_z} \\ -O_x^{A_z} & -O_y^{A_z} & 0 \end{pmatrix}$	$\begin{pmatrix} 0 & -O_x^{A_y} & 0 \\ -O_x^{A_y} & 0 & -O_z^{A_y} \\ 0 & -O_z^{A_y} & 0 \end{pmatrix}$
$(\frac{1}{2},0,\frac{1}{2})$	N/A	$\begin{pmatrix} 0 & -O_y^{A_x} & -O_z^{A_x} \\ -O_y^{A_x} & 0 & 0 \\ -O_z^{A_x} & 0 & 0 \end{pmatrix}$
$(\frac{1}{2},\frac{1}{2},0)$	$\begin{pmatrix} 0 & -O_y^{A_x} & -O_z^{A_x} \\ -O_y^{A_x} & 0 & 0 \\ -O_z^{A_x} & 0 & 0 \end{pmatrix}$	N/A

Supplementary Table 8. **The anti-symmetric exchange interaction $A'_{\tau\kappa}$ between the dipoles centered on $\mathbf{R}_m + \mathbf{r}_\tau$ and $\mathbf{R}_m + \mathbf{r}_\kappa$.** The tensor $A'_{\tau\kappa}$ is derived with respect to H_4.

$\mathbf{r}_\tau$ \ $\mathbf{r}_\kappa$	$(0, 0, 0)$	$(0, \frac{1}{2}, \frac{1}{2})$
$(0, 0, 0)$	N/A	$\begin{pmatrix} 0 & O_x^{A_y} & O_x^{A_z} \\ -O_x^{A_y} & 0 & O_z^{A_y} - O_y^{A_z} \\ -O_x^{A_z} & O_y^{A_z} - O_z^{A_y} & 0 \end{pmatrix}$
$(0, \frac{1}{2}, \frac{1}{2})$	$\begin{pmatrix} 0 & -O_x^{A_y} & -O_x^{A_z} \\ O_x^{A_y} & 0 & O_y^{A_z} - O_z^{A_y} \\ O_x^{A_z} & O_z^{A_y} - O_y^{A_z} & 0 \end{pmatrix}$	N/A
$(\frac{1}{2}, 0, \frac{1}{2})$	$\begin{pmatrix} 0 & O_y^{A_x} & O_x^{A_z} - O_z^{A_x} \\ -O_y^{A_x} & 0 & -O_y^{A_z} \\ O_z^{A_x} - O_x^{A_z} & O_y^{A_z} & 0 \end{pmatrix}$	$\begin{pmatrix} 0 & -O_y^{A_x} - O_x^{A_y} & O_z^{A_x} \\ O_y^{A_x} + O_x^{A_y} & 0 & -O_z^{A_y} \\ -O_z^{A_x} & O_z^{A_y} & 0 \end{pmatrix}$
$(\frac{1}{2}, \frac{1}{2}, 0)$	$\begin{pmatrix} 0 & O_x^{A_y} - O_y^{A_x} & O_z^{A_x} \\ O_y^{A_x} - O_x^{A_y} & 0 & O_z^{A_y} \\ -O_z^{A_x} & -O_z^{A_y} & 0 \end{pmatrix}$	$\begin{pmatrix} 0 & O_y^{A_x} & -O_z^{A_x} - O_x^{A_z} \\ -O_y^{A_x} & 0 & O_y^{A_z} \\ O_z^{A_x} + O_x^{A_z} & -O_y^{A_z} & 0 \end{pmatrix}$

$\mathbf{r}_\tau$ \ $\mathbf{r}_\kappa$	$(\frac{1}{2}, 0, \frac{1}{2})$	$(\frac{1}{2}, \frac{1}{2}, 0)$
$(0, 0, 0)$	$\begin{pmatrix} 0 & -O_y^{A_x} & O_z^{A_x} - O_x^{A_z} \\ O_y^{A_x} & 0 & O_y^{A_z} \\ O_x^{A_z} - O_z^{A_x} & -O_y^{A_z} & 0 \end{pmatrix}$	$\begin{pmatrix} 0 & O_y^{A_x} - O_x^{A_y} & -O_z^{A_x} \\ O_x^{A_y} - O_y^{A_x} & 0 & -O_z^{A_y} \\ O_z^{A_x} & O_z^{A_y} & 0 \end{pmatrix}$
$(0, \frac{1}{2}, \frac{1}{2})$	$\begin{pmatrix} 0 & O_y^{A_x} + O_x^{A_y} & -O_z^{A_x} \\ -O_y^{A_x} - O_x^{A_y} & 0 & O_z^{A_y} \\ O_z^{A_x} & -O_z^{A_y} & 0 \end{pmatrix}$	$\begin{pmatrix} 0 & -O_y^{A_x} & O_z^{A_x} + O_x^{A_z} \\ O_y^{A_x} & 0 & -O_y^{A_z} \\ -O_z^{A_x} - O_x^{A_z} & O_y^{A_z} & 0 \end{pmatrix}$
$(\frac{1}{2}, 0, \frac{1}{2})$	N/A	$\begin{pmatrix} 0 & O_x^{A_y} & -O_x^{A_z} \\ -O_x^{A_y} & 0 & O_z^{A_y} + O_y^{A_z} \\ O_x^{A_z} & -O_z^{A_y} - O_y^{A_z} & 0 \end{pmatrix}$
$(\frac{1}{2}, \frac{1}{2}, 0)$	$\begin{pmatrix} 0 & -O_x^{A_y} & O_x^{A_z} \\ O_x^{A_y} & 0 & -O_z^{A_y} - O_y^{A_z} \\ -O_x^{A_z} & O_z^{A_y} + O_y^{A_z} & 0 \end{pmatrix}$	N/A

Supplementary Table 9. **The anisotropic symmetric exchange interaction $S'_{\tau\kappa}$ between the dipoles centered on $\mathbf{R}_m + \mathbf{r}_\tau$ and $\mathbf{R}_m + \mathbf{r}_\kappa$.** The tensor $S'_{\tau\kappa}$ is derived with respect to H_4.

$\mathbf{r}_\tau$ \ $\mathbf{r}_\kappa$	$(0,0,0)$	$(0,\frac{1}{2},\frac{1}{2})$
$(0,0,0)$	N/A	$\begin{pmatrix} 0 & -O_y^{A_x} & -O_z^{A_x} \\ -O_y^{A_x} & 0 & 0 \\ -O_z^{A_x} & 0 & 0 \end{pmatrix}$
$(0,\frac{1}{2},\frac{1}{2})$	$\begin{pmatrix} 0 & -O_y^{A_x} & -O_z^{A_x} \\ -O_y^{A_x} & 0 & 0 \\ -O_z^{A_x} & 0 & 0 \end{pmatrix}$	N/A
$(\frac{1}{2},0,\frac{1}{2})$	$\begin{pmatrix} 0 & -O_x^{A_y} & 0 \\ -O_x^{A_y} & 0 & -O_z^{A_y} \\ 0 & -O_z^{A_y} & 0 \end{pmatrix}$	$\begin{pmatrix} 0 & 0 & O_x^{A_z} \\ 0 & 0 & O_y^{A_z} \\ O_x^{A_z} & O_y^{A_z} & 0 \end{pmatrix}$
$(\frac{1}{2},\frac{1}{2},0)$	$\begin{pmatrix} 0 & 0 & -O_x^{A_z} \\ 0 & 0 & -O_y^{A_z} \\ -O_x^{A_z} & -O_y^{A_z} & 0 \end{pmatrix}$	$\begin{pmatrix} 0 & O_x^{A_y} & 0 \\ O_x^{A_y} & 0 & O_z^{A_y} \\ 0 & O_z^{A_y} & 0 \end{pmatrix}$

$\mathbf{r}_\tau$ \ $\mathbf{r}_\kappa$	$(\frac{1}{2},0,\frac{1}{2})$	$(\frac{1}{2},\frac{1}{2},0)$
$(0,0,0)$	$\begin{pmatrix} 0 & -O_x^{A_y} & 0 \\ -O_x^{A_y} & 0 & -O_z^{A_y} \\ 0 & -O_z^{A_y} & 0 \end{pmatrix}$	$\begin{pmatrix} 0 & 0 & -O_x^{A_z} \\ 0 & 0 & -O_y^{A_z} \\ -O_x^{A_z} & -O_y^{A_z} & 0 \end{pmatrix}$
$(0,\frac{1}{2},\frac{1}{2})$	$\begin{pmatrix} 0 & 0 & O_x^{A_z} \\ 0 & 0 & O_y^{A_z} \\ O_x^{A_z} & O_y^{A_z} & 0 \end{pmatrix}$	$\begin{pmatrix} 0 & O_x^{A_y} & 0 \\ O_x^{A_y} & 0 & O_z^{A_y} \\ 0 & O_z^{A_y} & 0 \end{pmatrix}$
$(\frac{1}{2},0,\frac{1}{2})$	N/A	$\begin{pmatrix} 0 & O_y^{A_x} & O_z^{A_x} \\ O_y^{A_x} & 0 & 0 \\ O_z^{A_x} & 0 & 0 \end{pmatrix}$
$(\frac{1}{2},\frac{1}{2},0)$	$\begin{pmatrix} 0 & O_y^{A_x} & O_z^{A_x} \\ O_y^{A_x} & 0 & 0 \\ O_z^{A_x} & 0 & 0 \end{pmatrix}$	N/A

Supplementary Table 10. **The anisotropic symmetric exchange interaction $S'_{\tau\kappa}$ between the dipoles centered on $\mathbf{R}_m + \mathbf{r}_\tau$ and $\mathbf{R}_m + \mathbf{r}_\kappa$.** The tensor $S'_{\tau\kappa}$ is derived with respect to H_5.

$\mathbf{r}_\tau$ \ $\mathbf{r}_\kappa$	$(0,0,0)$	$(0,\frac{1}{2},\frac{1}{2})$
$(0,0,0)$	N/A	$\begin{pmatrix} 0 & O_z^G & O_y^G \\ O_z^G & 0 & O_x^G \\ O_y^G & O_x^G & 0 \end{pmatrix}$
$(0,\frac{1}{2},\frac{1}{2})$	$\begin{pmatrix} 0 & O_z^G & O_y^G \\ O_z^G & 0 & O_x^G \\ O_y^G & O_x^G & 0 \end{pmatrix}$	N/A
$(\frac{1}{2},0,\frac{1}{2})$	$\begin{pmatrix} 0 & O_z^G & O_y^G \\ O_z^G & 0 & O_x^G \\ O_y^G & O_x^G & 0 \end{pmatrix}$	$\begin{pmatrix} 0 & O_z^G & O_y^G \\ O_z^G & 0 & O_x^G \\ O_y^G & O_x^G & 0 \end{pmatrix}$
$(\frac{1}{2},\frac{1}{2},0)$	$\begin{pmatrix} 0 & O_z^G & O_y^G \\ O_z^G & 0 & O_x^G \\ O_y^G & O_x^G & 0 \end{pmatrix}$	$\begin{pmatrix} 0 & O_z^G & O_y^G \\ O_z^G & 0 & O_x^G \\ O_y^G & O_x^G & 0 \end{pmatrix}$

$\mathbf{r}_\tau$ \ $\mathbf{r}_\kappa$	$(\frac{1}{2},0,\frac{1}{2})$	$(\frac{1}{2},\frac{1}{2},0)$
$(0,0,0)$	$\begin{pmatrix} 0 & O_z^G & O_y^G \\ O_z^G & 0 & O_x^G \\ O_y^G & O_x^G & 0 \end{pmatrix}$	$\begin{pmatrix} 0 & O_z^G & O_y^G \\ O_z^G & 0 & O_x^G \\ O_y^G & O_x^G & 0 \end{pmatrix}$
$(0,\frac{1}{2},\frac{1}{2})$	$\begin{pmatrix} 0 & O_z^G & O_y^G \\ O_z^G & 0 & O_x^G \\ O_y^G & O_x^G & 0 \end{pmatrix}$	$\begin{pmatrix} 0 & O_z^G & O_y^G \\ O_z^G & 0 & O_x^G \\ O_y^G & O_x^G & 0 \end{pmatrix}$
$(\frac{1}{2},0,\frac{1}{2})$	N/A	$\begin{pmatrix} 0 & O_z^G & O_y^G \\ O_z^G & 0 & O_x^G \\ O_y^G & O_x^G & 0 \end{pmatrix}$
$(\frac{1}{2},\frac{1}{2},0)$	$\begin{pmatrix} 0 & O_z^G & O_y^G \\ O_z^G & 0 & O_x^G \\ O_y^G & O_x^G & 0 \end{pmatrix}$	N/A

Supplementary Table 11. **The anisotropic symmetric exchange interaction $S'_{\tau\kappa}$ between the dipoles centered on $\mathbf{R}_m + \mathbf{r}_\tau$ and $\mathbf{R}_m + \mathbf{r}_\kappa$.** The tensor $S'_{\tau\kappa}$ is derived with respect to H_6.

$\mathbf{r}_\tau$ \ $\mathbf{r}_\kappa$	$(0,0,0)$	$(0,\frac{1}{2},\frac{1}{2})$	$(\frac{1}{2},0,\frac{1}{2})$	$(\frac{1}{2},\frac{1}{2},0)$
$(0,0,0)$	N/A	$\begin{pmatrix} 0 & -O_z^G & -O_y^G \\ -O_z^G & 0 & O_x^G \\ -O_y^G & O_x^G & 0 \end{pmatrix}$	$\begin{pmatrix} 0 & -O_z^G & O_y^G \\ -O_z^G & 0 & -O_x^G \\ O_y^G & -O_x^G & 0 \end{pmatrix}$	$\begin{pmatrix} 0 & O_z^G & -O_y^G \\ O_z^G & 0 & -O_x^G \\ -O_y^G & -O_x^G & 0 \end{pmatrix}$
$(0,\frac{1}{2},\frac{1}{2})$	$\begin{pmatrix} 0 & -O_z^G & -O_y^G \\ -O_z^G & 0 & O_x^G \\ -O_y^G & O_x^G & 0 \end{pmatrix}$	N/A	$\begin{pmatrix} 0 & O_z^G & -O_y^G \\ O_z^G & 0 & -O_x^G \\ -O_y^G & -O_x^G & 0 \end{pmatrix}$	$\begin{pmatrix} 0 & -O_z^G & O_y^G \\ -O_z^G & 0 & -O_x^G \\ O_y^G & -O_x^G & 0 \end{pmatrix}$
$(\frac{1}{2},0,\frac{1}{2})$	$\begin{pmatrix} 0 & -O_z^G & O_y^G \\ -O_z^G & 0 & -O_x^G \\ O_y^G & -O_x^G & 0 \end{pmatrix}$	$\begin{pmatrix} 0 & O_z^G & -O_y^G \\ O_z^G & 0 & -O_x^G \\ -O_y^G & -O_x^G & 0 \end{pmatrix}$	N/A	$\begin{pmatrix} 0 & -O_z^G & -O_y^G \\ -O_z^G & 0 & O_x^G \\ -O_y^G & O_x^G & 0 \end{pmatrix}$
$(\frac{1}{2},\frac{1}{2},0)$	$\begin{pmatrix} 0 & O_z^G & -O_y^G \\ O_z^G & 0 & -O_x^G \\ -O_y^G & -O_x^G & 0 \end{pmatrix}$	$\begin{pmatrix} 0 & -O_z^G & O_y^G \\ -O_z^G & 0 & -O_x^G \\ O_y^G & -O_x^G & 0 \end{pmatrix}$	$\begin{pmatrix} 0 & -O_z^G & -O_y^G \\ -O_z^G & 0 & O_x^G \\ -O_y^G & O_x^G & 0 \end{pmatrix}$	N/A

Supplementary Table 12. **The anisotropic symmetric exchange interaction $S'_{\tau\kappa}$ between the dipoles centered on $\mathbf{R}_m + \mathbf{r}_\tau$ and $\mathbf{R}_m + \mathbf{r}_\kappa$.** The tensor $S'_{\tau\kappa}$ is derived with respect to H_7.

$\mathbf{r}_\tau$ \ $\mathbf{r}_\kappa$	$(0,0,0)$	$(0,\frac{1}{2},\frac{1}{2})$
$(0,0,0)$	N/A	$\begin{pmatrix} 0 & 0 & 0 \\ 0 & 0 & -2O_x^G \\ 0 & -2O_x^G & 0 \end{pmatrix}$
$(0,\frac{1}{2},\frac{1}{2})$	$\begin{pmatrix} 0 & 0 & 0 \\ 0 & 0 & -2O_x^G \\ 0 & -2O_x^G & 0 \end{pmatrix}$	N/A
$(\frac{1}{2},0,\frac{1}{2})$	$\begin{pmatrix} 0 & 0 & -2O_y^G \\ 0 & 0 & 0 \\ -2O_y^G & 0 & 0 \end{pmatrix}$	$\begin{pmatrix} 0 & -2O_z^G & 0 \\ -2O_z^G & 0 & 0 \\ 0 & 0 & 0 \end{pmatrix}$
$(\frac{1}{2},\frac{1}{2},0)$	$\begin{pmatrix} 0 & -2O_z^G & 0 \\ -2O_z^G & 0 & 0 \\ 0 & 0 & 0 \end{pmatrix}$	$\begin{pmatrix} 0 & 0 & -2O_y^G \\ 0 & 0 & 0 \\ -2O_y^G & 0 & 0 \end{pmatrix}$

$\mathbf{r}_\tau$ \ $\mathbf{r}_\kappa$	$(\frac{1}{2},0,\frac{1}{2})$	$(\frac{1}{2},\frac{1}{2},0)$
$(0,0,0)$	$\begin{pmatrix} 0 & 0 & -2O_y^G \\ 0 & 0 & 0 \\ -2O_y^G & 0 & 0 \end{pmatrix}$	$\begin{pmatrix} 0 & -2O_z^G & 0 \\ -2O_z^G & 0 & 0 \\ 0 & 0 & 0 \end{pmatrix}$
$(0,\frac{1}{2},\frac{1}{2})$	$\begin{pmatrix} 0 & -2O_z^G & 0 \\ -2O_z^G & 0 & 0 \\ 0 & 0 & 0 \end{pmatrix}$	$\begin{pmatrix} 0 & 0 & -2O_y^G \\ 0 & 0 & 0 \\ -2O_y^G & 0 & 0 \end{pmatrix}$
$(\frac{1}{2},0,\frac{1}{2})$	N/A	$\begin{pmatrix} 0 & 0 & 0 \\ 0 & 0 & -2O_x^G \\ 0 & -2O_x^G & 0 \end{pmatrix}$
$(\frac{1}{2},\frac{1}{2},0)$	$\begin{pmatrix} 0 & 0 & 0 \\ 0 & 0 & -2O_x^G \\ 0 & -2O_x^G & 0 \end{pmatrix}$	N/A

Supplementary Table 13. **The anisotropic symmetric exchange interaction $S'_{\tau\kappa}$ between the dipoles centered on $\mathbf{R}_m + \mathbf{r}_\tau$ and $\mathbf{R}_m + \mathbf{r}_\kappa$.** The tensor $S'_{\tau\kappa}$ is derived with respect to H_8.

$\mathbf{r}_\tau$ \ $\mathbf{r}_\kappa$	$(0,0,0)$	$(0,\frac{1}{2},\frac{1}{2})$
$(0,0,0)$	N/A	$\begin{pmatrix} -\frac{4O_x^{Ax}}{3} & 0 & 0 \\ 0 & \frac{2O_x^{Ax}}{3} & 0 \\ 0 & 0 & \frac{2O_x^{Ax}}{3} \end{pmatrix}$
$(0,\frac{1}{2},\frac{1}{2})$	$\begin{pmatrix} -\frac{4O_x^{Ax}}{3} & 0 & 0 \\ 0 & \frac{2O_x^{Ax}}{3} & 0 \\ 0 & 0 & \frac{2O_x^{Ax}}{3} \end{pmatrix}$	N/A
$(\frac{1}{2},0,\frac{1}{2})$	$\begin{pmatrix} \frac{2O_y^{Ay}}{3} & 0 & 0 \\ 0 & -\frac{4O_y^{Ay}}{3} & 0 \\ 0 & 0 & \frac{2O_y^{Ay}}{3} \end{pmatrix}$	$\begin{pmatrix} -\frac{2O_z^{Az}}{3} & 0 & 0 \\ 0 & -\frac{2O_z^{Az}}{3} & 0 \\ 0 & 0 & \frac{4O_z^{Az}}{3} \end{pmatrix}$
$(\frac{1}{2},\frac{1}{2},0)$	$\begin{pmatrix} \frac{2O_z^{Az}}{3} & 0 & 0 \\ 0 & \frac{2O_z^{Az}}{3} & 0 \\ 0 & 0 & -\frac{4O_z^{Az}}{3} \end{pmatrix}$	$\begin{pmatrix} -\frac{2O_y^{Ay}}{3} & 0 & 0 \\ 0 & \frac{4O_y^{Ay}}{3} & 0 \\ 0 & 0 & -\frac{2O_y^{Ay}}{3} \end{pmatrix}$

$\mathbf{r}_\tau$ \ $\mathbf{r}_\kappa$	$(\frac{1}{2},0,\frac{1}{2})$	$(\frac{1}{2},\frac{1}{2},0)$
$(0,0,0)$	$\begin{pmatrix} \frac{2O_y^{Ay}}{3} & 0 & 0 \\ 0 & -\frac{4O_y^{Ay}}{3} & 0 \\ 0 & 0 & \frac{2O_y^{Ay}}{3} \end{pmatrix}$	$\begin{pmatrix} \frac{2O_z^{Az}}{3} & 0 & 0 \\ 0 & \frac{2O_z^{Az}}{3} & 0 \\ 0 & 0 & -\frac{4O_z^{Az}}{3} \end{pmatrix}$
$(0,\frac{1}{2},\frac{1}{2})$	$\begin{pmatrix} -\frac{2O_z^{Az}}{3} & 0 & 0 \\ 0 & -\frac{2O_z^{Az}}{3} & 0 \\ 0 & 0 & \frac{4O_z^{Az}}{3} \end{pmatrix}$	$\begin{pmatrix} -\frac{2O_y^{Ay}}{3} & 0 & 0 \\ 0 & \frac{4O_y^{Ay}}{3} & 0 \\ 0 & 0 & -\frac{2O_y^{Ay}}{3} \end{pmatrix}$
$(\frac{1}{2},0,\frac{1}{2})$	N/A	$\begin{pmatrix} \frac{4O_x^{Ax}}{3} & 0 & 0 \\ 0 & -\frac{2O_x^{Ax}}{3} & 0 \\ 0 & 0 & -\frac{2O_x^{Ax}}{3} \end{pmatrix}$
$(\frac{1}{2},\frac{1}{2},0)$	$\begin{pmatrix} \frac{4O_x^{Ax}}{3} & 0 & 0 \\ 0 & -\frac{2O_x^{Ax}}{3} & 0 \\ 0 & 0 & -\frac{2O_x^{Ax}}{3} \end{pmatrix}$	N/A

Supplementary Table 14. **The anisotropic symmetric exchange interaction $S'_{\tau\kappa}$ between the dipoles centered on $\mathbf{R}_m + \mathbf{r}_\tau$ and $\mathbf{R}_m + \mathbf{r}_\kappa$.** The tensor $S'_{\tau\kappa}$ is derived with respect to H_9.

$\mathbf{r}_\tau$ \ $\mathbf{r}_\kappa$	$(0,0,0)$	$(0,\frac{1}{2},\frac{1}{2})$
$(0,0,0)$	N/A	$\begin{pmatrix} \frac{4O_x^{Ax}}{3} & 0 & 0 \\ 0 & -\frac{2O_x^{Ax}}{3} & 0 \\ 0 & 0 & -\frac{2O_x^{Ax}}{3} \end{pmatrix}$
$(0,\frac{1}{2},\frac{1}{2})$	$\begin{pmatrix} \frac{4O_x^{Ax}}{3} & 0 & 0 \\ 0 & -\frac{2O_x^{Ax}}{3} & 0 \\ 0 & 0 & -\frac{2O_x^{Ax}}{3} \end{pmatrix}$	N/A
$(\frac{1}{2},0,\frac{1}{2})$	$\begin{pmatrix} -\frac{2O_y^{Ay}}{3} & 0 & 0 \\ 0 & \frac{4O_y^{Ay}}{3} & 0 \\ 0 & 0 & -\frac{2O_y^{Ay}}{3} \end{pmatrix}$	$\begin{pmatrix} \frac{2O_z^{Az}}{3} & 0 & 0 \\ 0 & \frac{2O_z^{Az}}{3} & 0 \\ 0 & 0 & -\frac{4O_z^{Az}}{3} \end{pmatrix}$
$(\frac{1}{2},\frac{1}{2},0)$	$\begin{pmatrix} -\frac{2O_z^{Az}}{3} & 0 & 0 \\ 0 & -\frac{2O_z^{Az}}{3} & 0 \\ 0 & 0 & \frac{4O_z^{Az}}{3} \end{pmatrix}$	$\begin{pmatrix} \frac{2O_y^{Ay}}{3} & 0 & 0 \\ 0 & -\frac{4O_y^{Ay}}{3} & 0 \\ 0 & 0 & \frac{2O_y^{Ay}}{3} \end{pmatrix}$

$\mathbf{r}_\tau$ \ $\mathbf{r}_\kappa$	$(\frac{1}{2},0,\frac{1}{2})$	$(\frac{1}{2},\frac{1}{2},0)$
$(0,0,0)$	$\begin{pmatrix} -\frac{2O_y^{Ay}}{3} & 0 & 0 \\ 0 & \frac{4O_y^{Ay}}{3} & 0 \\ 0 & 0 & -\frac{2O_y^{Ay}}{3} \end{pmatrix}$	$\begin{pmatrix} -\frac{2O_z^{Az}}{3} & 0 & 0 \\ 0 & -\frac{2O_z^{Az}}{3} & 0 \\ 0 & 0 & \frac{4O_z^{Az}}{3} \end{pmatrix}$
$(0,\frac{1}{2},\frac{1}{2})$	$\begin{pmatrix} \frac{2O_z^{Az}}{3} & 0 & 0 \\ 0 & \frac{2O_z^{Az}}{3} & 0 \\ 0 & 0 & -\frac{4O_z^{Az}}{3} \end{pmatrix}$	$\begin{pmatrix} \frac{2O_y^{Ay}}{3} & 0 & 0 \\ 0 & -\frac{4O_y^{Ay}}{3} & 0 \\ 0 & 0 & \frac{2O_y^{Ay}}{3} \end{pmatrix}$
$(\frac{1}{2},0,\frac{1}{2})$	N/A	$\begin{pmatrix} -\frac{4O_x^{Ax}}{3} & 0 & 0 \\ 0 & \frac{2O_x^{Ax}}{3} & 0 \\ 0 & 0 & \frac{2O_x^{Ax}}{3} \end{pmatrix}$
$(\frac{1}{2},\frac{1}{2},0)$	$\begin{pmatrix} -\frac{4O_x^{Ax}}{3} & 0 & 0 \\ 0 & \frac{2O_x^{Ax}}{3} & 0 \\ 0 & 0 & \frac{2O_x^{Ax}}{3} \end{pmatrix}$	N/A

Supplementary Table 15. **The anisotropic symmetric exchange interaction $S'_{\tau\kappa}$ between the dipoles centered on $\mathbf{R}_m + \mathbf{r}_\tau$ and $\mathbf{R}_m + \mathbf{r}_\kappa$.** The tensor $S'_{\tau\kappa}$ is derived with respect to H_{10}.

$\mathbf{r}_\tau$ \ $\mathbf{r}_\kappa$	$(0, 0, 0)$	$(0, \frac{1}{2}, \frac{1}{2})$
$(0, 0, 0)$	N/A	$\begin{pmatrix} \frac{4O_x^{Ax}}{3} & 0 & 0 \\ 0 & -\frac{2O_x^{Ax}}{3} & 0 \\ 0 & 0 & -\frac{2O_x^{Ax}}{3} \end{pmatrix}$
$(0, \frac{1}{2}, \frac{1}{2})$	$\begin{pmatrix} \frac{4O_x^{Ax}}{3} & 0 & 0 \\ 0 & -\frac{2O_x^{Ax}}{3} & 0 \\ 0 & 0 & -\frac{2O_x^{Ax}}{3} \end{pmatrix}$	N/A
$(\frac{1}{2}, 0, \frac{1}{2})$	$\begin{pmatrix} -\frac{2O_y^{Ay}}{3} & 0 & 0 \\ 0 & \frac{4O_y^{Ay}}{3} & 0 \\ 0 & 0 & -\frac{2O_y^{Ay}}{3} \end{pmatrix}$	$\begin{pmatrix} \frac{2O_z^{Az}}{3} & 0 & 0 \\ 0 & \frac{2O_z^{Az}}{3} & 0 \\ 0 & 0 & -\frac{4O_z^{Az}}{3} \end{pmatrix}$
$(\frac{1}{2}, \frac{1}{2}, 0)$	$\begin{pmatrix} -\frac{2O_z^{Az}}{3} & 0 & 0 \\ 0 & -\frac{2O_z^{Az}}{3} & 0 \\ 0 & 0 & \frac{4O_z^{Az}}{3} \end{pmatrix}$	$\begin{pmatrix} \frac{2O_y^{Ay}}{3} & 0 & 0 \\ 0 & -\frac{4O_y^{Ay}}{3} & 0 \\ 0 & 0 & \frac{2O_y^{Ay}}{3} \end{pmatrix}$

$\mathbf{r}_\tau$ \ $\mathbf{r}_\kappa$	$(\frac{1}{2}, 0, \frac{1}{2})$	$(\frac{1}{2}, \frac{1}{2}, 0)$
$(0, 0, 0)$	$\begin{pmatrix} -\frac{2O_y^{Ay}}{3} & 0 & 0 \\ 0 & \frac{4O_y^{Ay}}{3} & 0 \\ 0 & 0 & -\frac{2O_y^{Ay}}{3} \end{pmatrix}$	$\begin{pmatrix} -\frac{2O_z^{Az}}{3} & 0 & 0 \\ 0 & -\frac{2O_z^{Az}}{3} & 0 \\ 0 & 0 & \frac{4O_z^{Az}}{3} \end{pmatrix}$
$(0, \frac{1}{2}, \frac{1}{2})$	$\begin{pmatrix} \frac{2O_z^{Az}}{3} & 0 & 0 \\ 0 & \frac{2O_z^{Az}}{3} & 0 \\ 0 & 0 & -\frac{4O_z^{Az}}{3} \end{pmatrix}$	$\begin{pmatrix} \frac{2O_y^{Ay}}{3} & 0 & 0 \\ 0 & -\frac{4O_y^{Ay}}{3} & 0 \\ 0 & 0 & \frac{2O_y^{Ay}}{3} \end{pmatrix}$
$(\frac{1}{2}, 0, \frac{1}{2})$	N/A	$\begin{pmatrix} -\frac{4O_x^{Ax}}{3} & 0 & 0 \\ 0 & \frac{2O_x^{Ax}}{3} & 0 \\ 0 & 0 & \frac{2O_x^{Ax}}{3} \end{pmatrix}$
$(\frac{1}{2}, \frac{1}{2}, 0)$	$\begin{pmatrix} -\frac{4O_x^{Ax}}{3} & 0 & 0 \\ 0 & \frac{2O_x^{Ax}}{3} & 0 \\ 0 & 0 & \frac{2O_x^{Ax}}{3} \end{pmatrix}$	N/A

Supplementary Table 16. **The anisotropic symmetric exchange interaction $S'_{\tau\kappa}$ between the dipoles centered on $\mathbf{R}_m + \mathbf{r}_\tau$ and $\mathbf{R}_m + \mathbf{r}_\kappa$.** The tensor $S'_{\tau\kappa}$ is derived with respect to H_{11}.

$\mathbf{r}_\tau$ \ $\mathbf{r}_\kappa$	$(0,0,0)$	$(0,\frac{1}{2},\frac{1}{2})$
$(0,0,0)$	N/A	$\begin{pmatrix} -\frac{4O_x^{A_x}}{3} & 0 & 0 \\ 0 & \frac{2O_x^{A_x}}{3} & 0 \\ 0 & 0 & \frac{2O_x^{A_x}}{3} \end{pmatrix}$
$(0,\frac{1}{2},\frac{1}{2})$	$\begin{pmatrix} -\frac{4O_x^{A_x}}{3} & 0 & 0 \\ 0 & \frac{2O_x^{A_x}}{3} & 0 \\ 0 & 0 & \frac{2O_x^{A_x}}{3} \end{pmatrix}$	N/A
$(\frac{1}{2},0,\frac{1}{2})$	$\begin{pmatrix} \frac{2O_y^{A_y}}{3} & 0 & 0 \\ 0 & -\frac{4O_y^{A_y}}{3} & 0 \\ 0 & 0 & \frac{2O_y^{A_y}}{3} \end{pmatrix}$	$\begin{pmatrix} -\frac{2O_z^{A_z}}{3} & 0 & 0 \\ 0 & -\frac{2O_z^{A_z}}{3} & 0 \\ 0 & 0 & \frac{4O_z^{A_z}}{3} \end{pmatrix}$
$(\frac{1}{2},\frac{1}{2},0)$	$\begin{pmatrix} \frac{2O_z^{A_z}}{3} & 0 & 0 \\ 0 & \frac{2O_z^{A_z}}{3} & 0 \\ 0 & 0 & -\frac{4O_z^{A_z}}{3} \end{pmatrix}$	$\begin{pmatrix} -\frac{2O_y^{A_y}}{3} & 0 & 0 \\ 0 & \frac{4O_y^{A_y}}{3} & 0 \\ 0 & 0 & -\frac{2O_y^{A_y}}{3} \end{pmatrix}$

$\mathbf{r}_\tau$ \ $\mathbf{r}_\kappa$	$(\frac{1}{2},0,\frac{1}{2})$	$(\frac{1}{2},\frac{1}{2},0)$
$(0,0,0)$	$\begin{pmatrix} \frac{2O_y^{A_y}}{3} & 0 & 0 \\ 0 & -\frac{4O_y^{A_y}}{3} & 0 \\ 0 & 0 & \frac{2O_y^{A_y}}{3} \end{pmatrix}$	$\begin{pmatrix} \frac{2O_z^{A_z}}{3} & 0 & 0 \\ 0 & \frac{2O_z^{A_z}}{3} & 0 \\ 0 & 0 & -\frac{4O_z^{A_z}}{3} \end{pmatrix}$
$(0,\frac{1}{2},\frac{1}{2})$	$\begin{pmatrix} -\frac{2O_z^{A_z}}{3} & 0 & 0 \\ 0 & -\frac{2O_z^{A_z}}{3} & 0 \\ 0 & 0 & \frac{4O_z^{A_z}}{3} \end{pmatrix}$	$\begin{pmatrix} -\frac{2O_y^{A_y}}{3} & 0 & 0 \\ 0 & \frac{4O_y^{A_y}}{3} & 0 \\ 0 & 0 & -\frac{2O_y^{A_y}}{3} \end{pmatrix}$
$(\frac{1}{2},0,\frac{1}{2})$	N/A	$\begin{pmatrix} \frac{4O_x^{A_x}}{3} & 0 & 0 \\ 0 & -\frac{2O_x^{A_x}}{3} & 0 \\ 0 & 0 & -\frac{2O_x^{A_x}}{3} \end{pmatrix}$
$(\frac{1}{2},\frac{1}{2},0)$	$\begin{pmatrix} \frac{4O_x^{A_x}}{3} & 0 & 0 \\ 0 & -\frac{2O_x^{A_x}}{3} & 0 \\ 0 & 0 & -\frac{2O_x^{A_x}}{3} \end{pmatrix}$	N/A

Supplementary Table 17. **The $A'_{\tau\kappa}$ and $S'_{\tau\kappa}$ exchange interactions in hafnia.** As Table 2 in the Main Text, but arising from $O_y^{A_x}$.

(Hf$_\tau$,Hf$_\kappa$)	$A'_{\tau\kappa,\alpha\beta}$ or $S'_{\tau\kappa,\alpha\beta}$	(Hf$_\tau$,Hf$_\kappa$)	$A'_{\tau\kappa,\alpha\beta}$ or $S'_{\tau\kappa,\alpha\beta}$	(Hf$_\tau$,Hf$_\kappa$)	$A'_{\tau\kappa,\alpha\beta}$ or $S'_{\tau\kappa,\alpha\beta}$
(Hf_1, Hf_2)	$S'_{12,xy} = S'_{12,yx} = \alpha_3 O_y^{A_x}$	(Hf_2, Hf_3)	$A'_{23,xy} = -A'_{23,yx} = -\alpha_1 O_y^{A_x}$	(Hf_3, Hf_4)	$S'_{34,xy} = S'_{34,yx} = -\alpha_3 O_y^{A_x}$
(Hf_1, Hf_3)	$A'_{13,xy} = -A'_{13,yx} = -\alpha_2 O_y^{A_x}$	(Hf_2, Hf_4)	$A'_{24,xy} = -A'_{24,yx} = -\alpha_2 O_y^{A_x}$	(Hf_4, Hf_1)	$A'_{41,xy} = -A'_{41,yx} = \alpha_1 O_y^{A_x}$
(Hf_1, Hf_4)	$A'_{14,xy} = -A'_{14,yx} = -\alpha_1 O_y^{A_x}$	(Hf_3, Hf_1)	$A'_{31,xy} = -A'_{31,yx} = \alpha_2 O_y^{A_x}$	(Hf_4, Hf_2)	$A'_{42,xy} = -A'_{42,yx} = \alpha_2 O_y^{A_x}$
(Hf_2, Hf_1)	$S'_{21,xy} = S'_{21,yx} = \alpha_3 O_y^{A_x}$	(Hf_3, Hf_2)	$A'_{32,xy} = -A'_{32,yx} = \alpha_1 O_y^{A_x}$	(Hf_4, Hf_3)	$S'_{43,xy} = S'_{43,yx} = -\alpha_3 O_y^{A_x}$

Supplementary Table 18. **The $A'_{\tau\kappa}$ and $S'_{\tau\kappa}$ exchange interactions in hafnia.** As Table 2 in the Main Text, but arising from $O_y^{A_z}$.

(Hf_τ,Hf_κ)	$A'_{\tau\kappa,\alpha\beta}$ or $S'_{ij,\alpha\beta}$	(Hf_τ,Hf_κ)	$A'_{\tau\kappa,\alpha\beta}$ or $S'_{ij,\alpha\beta}$	(Hf_τ,Hf_κ)	$A'_{\tau\kappa,\alpha\beta}$ or $S'_{ij,\alpha\beta}$
(Hf_1, Hf_2)	$A'_{12,yz} = -A'_{12,zy} = \alpha_1 O_y^{A_z}$	(Hf_2, Hf_3)	$S'_{23,yz} = S'_{23,zy} = -\alpha_3 O_y^{A_z}$	(Hf_3, Hf_4)	$A'_{34,yz} = -A'_{34,zy} = -\alpha_1 O_y^{A_z}$
(Hf_1, Hf_3)	$A'_{13,yz} = -A'_{13,zy} = \alpha_2 O_y^{A_z}$	(Hf_2, Hf_4)	$A'_{24,yz} = -A'_{24,zy} = -\alpha_2 O_y^{A_z}$	(Hf_4, Hf_1)	$S'_{41,yz} = S'_{41,zy} = \alpha_3 O_y^{A_z}$
(Hf_1, Hf_4)	$S'_{14,yz} = S'_{14,zy} = \alpha_3 O_y^{A_z}$	(Hf_3, Hf_1)	$A'_{31,yz} = -A'_{31,zy} = -\alpha_2 O_y^{A_z}$	(Hf_4, Hf_2)	$A'_{42,yz} = -A'_{42,zy} = \alpha_2 O_y^{A_z}$
(Hf_2, Hf_1)	$A'_{21,yz} = -A'_{21,zy} = -\alpha_1 O_y^{A_z}$	(Hf_3, Hf_2)	$S'_{32,yz} = S'_{32,zy} = -\alpha_3 O_y^{A_z}$	(Hf_4, Hf_3)	$A'_{43,yz} = -A'_{43,zy} = \alpha_1 O_y^{A_z}$

Supplementary Table 19. **The $A'_{\tau\kappa}$ and $S'_{\tau\kappa}$ exchange interactions in hafnia.** As Table 2 in the Main Text, but arising from $O_z^{A_y}$.

(Hf_τ,Hf_κ)	$A'_{\tau\kappa,\alpha\beta}$ or $S'_{ij,\alpha\beta}$	(Hf_τ,Hf_κ)	$A'_{\tau\kappa,\alpha\beta}$ or $S'_{ij,\alpha\beta}$	(Hf_τ,Hf_κ)	$A'_{\tau\kappa,\alpha\beta}$ or $S'_{ij,\alpha\beta}$
(Hf_1, Hf_2)	$A'_{12,yz} = -A'_{12,zy} = -\alpha_1 O_z^{A_y}$	(Hf_2, Hf_3)	$A'_{23,yz} = -A'_{23,zy} = \alpha_2 O_z^{A_y}$	(Hf_3, Hf_4)	$A'_{34,yz} = -A'_{34,zy} = -\alpha_1 O_z^{A_y}$
(Hf_1, Hf_3)	$S'_{13,yz} = S'_{13,zy} = \alpha_3 O_z^{A_y}$	(Hf_2, Hf_4)	$S'_{24,yz} = S'_{24,zy} = -\alpha_3 O_z^{A_y}$	(Hf_4, Hf_1)	$A'_{41,yz} = -A'_{41,zy} = \alpha_2 O_z^{A_y}$
(Hf_1, Hf_4)	$A'_{14,yz} = -A'_{14,zy} = -\alpha_2 O_z^{A_y}$	(Hf_3, Hf_1)	$S'_{31,yz} = S'_{31,zy} = \alpha_3 O_z^{A_y}$	(Hf_4, Hf_2)	$S'_{42,yz} = S'_{42,zy} = -\alpha_3 O_z^{A_y}$
(Hf_2, Hf_1)	$A'_{21,yz} = -A'_{21,zy} = \alpha_1 O_z^{A_y}$	(Hf_3, Hf_2)	$A'_{32,yz} = -A'_{32,zy} = -\alpha_2 O_z^{A_y}$	(Hf_4, Hf_3)	$A'_{43,yz} = -A'_{43,zy} = \alpha_1 O_z^{A_y}$

Supplementary Table 20. **The $A'_{\tau\kappa}$ and $S'_{\tau\kappa}$ exchange interactions in hafnia.** As Table 2 in the Main Text, but arising from $O_x^{A_z}$.

(Hf_τ,Hf_κ)	$A'_{\tau\kappa,\alpha\beta}$ or $S'_{ij,\alpha\beta}$	(Hf_τ,Hf_κ)	$A'_{\tau\kappa,\alpha\beta}$ or $S'_{ij,\alpha\beta}$	(Hf_τ,Hf_κ)	$A'_{\tau\kappa,\alpha\beta}$ or $S'_{ij,\alpha\beta}$
(Hf_1, Hf_2)	$A'_{12,xz} = -A'_{12,zx} = \alpha_2 O_x^{A_z}$	(Hf_2, Hf_3)	$S'_{23,xz} = S'_{23,zx} = -\alpha_3 O_x^{A_z}$	(Hf_3, Hf_4)	$A'_{34,xz} = -A'_{34,zx} = -\alpha_2 O_x^{A_z}$
(Hf_1, Hf_3)	$A'_{13,xz} = -A'_{13,zx} = \alpha_1 O_x^{A_z}$	(Hf_2, Hf_4)	$A'_{24,xz} = -A'_{24,zx} = -\alpha_1 O_x^{A_z}$	(Hf_4, Hf_1)	$S'_{41,xz} = S'_{41,zx} = \alpha_3 O_x^{A_z}$
(Hf_1, Hf_4)	$S'_{14,xz} = S'_{14,zx} = \alpha_3 O_x^{A_z}$	(Hf_3, Hf_1)	$A'_{31,xz} = -A'_{31,zx} = -\alpha_1 O_x^{A_z}$	(Hf_4, Hf_2)	$A'_{42,xz} = -A'_{42,zx} = \alpha_1 O_x^{A_z}$
(Hf_2, Hf_1)	$A'_{21,xz} = -A'_{21,zx} = -\alpha_2 O_x^{A_z}$	(Hf_3, Hf_2)	$S'_{32,xz} = S'_{32,zx} = -\alpha_3 O_x^{A_z}$	(Hf_4, Hf_3)	$A'_{43,xz} = -A'_{43,zx} = \alpha_2 O_x^{A_z}$

Supplementary Table 21. **Seven spatial symmetry operations.** The descriptions of these symmetry operations and their transformation effects on the $\mathbf{u}_i \equiv (u_{i,x}, u_{i,y}, u_{i,z})$ and $\mathbf{u}_j \equiv (u_{j,x}, u_{j,y}, u_{j,z})$ dipoles are shown in the table.

Notations	Symmetry operations	Transformation rules
$\bar{1}$	Inversion center located at k	$(u_{i,x}, u_{i,y}, u_{i,z}) \rightarrow (-u_{j,x}, -u_{j,y}, -u_{j,z})$,<br>$(u_{j,x}, u_{j,y}, u_{j,z}) \rightarrow (-u_{i,x}, -u_{i,y}, -u_{i,z})$
$\mathfrak{m}_x$	Mirror plane perpendicular to x axis (passing k)	$(u_{i,x}, u_{i,y}, u_{i,z}) \rightarrow (-u_{j,x}, u_{j,y}, u_{j,z})$,<br>$(u_{j,x}, u_{j,y}, u_{j,z}) \rightarrow (-u_{i,x}, u_{i,y}, u_{i,z})$
$\mathfrak{m}_y$	Mirror plane perpendicular to y axis (passing k)	$(u_{i,x}, u_{i,y}, u_{i,z}) \rightarrow (u_{i,x}, -u_{i,y}, u_{i,z})$,<br>$(u_{j,x}, u_{j,y}, u_{j,z}) \rightarrow (u_{j,x}, -u_{j,y}, u_{j,z})$
$\mathfrak{m}_z$	Mirror plane perpendicular to z axis (passing k)	$(u_{i,x}, u_{i,y}, u_{i,z}) \rightarrow (u_{i,x}, u_{i,y}, -u_{i,z})$,<br>$(u_{j,x}, u_{j,y}, u_{j,z}) \rightarrow (u_{j,x}, u_{j,y}, -u_{j,z})$
$\mathfrak{2}_y$	Rotation of π along y axis	$(u_{i,x}, u_{i,y}, u_{i,z}) \rightarrow (-u_{j,x}, u_{j,y}, -u_{j,z})$,<br>$(u_{j,x}, u_{j,y}, u_{j,z}) \rightarrow (-u_{i,x}, u_{i,y}, -u_{i,z})$
$\mathfrak{2}_z$	Rotation of π along z axis	$(u_{i,x}, u_{i,y}, u_{i,z}) \rightarrow (-u_{j,x}, -u_{j,y}, u_{j,z})$,<br>$(u_{j,x}, u_{j,y}, u_{j,z}) \rightarrow (-u_{i,x}, -u_{i,y}, u_{i,z})$
$n_x (n \geq 2)$	Rotation of $\theta = \frac{2\pi}{n}$ along x axis	$(u_{i,x}, u_{i,y}, u_{i,z}) \rightarrow (u_{i,x}, u_{i,y} cos\theta - u_{i,z} sin\theta, u_{i,y} sin\theta + u_{i,z} cos\theta)$,<br>$(u_{j,x}, u_{j,y}, u_{j,z}) \rightarrow (u_{j,x}, u_{j,y} cos\theta - u_{j,z} sin\theta, u_{j,y} sin\theta + u_{j,z} cos\theta)$

Supplementary Table 22. **The symmetry rules regarding the anti-symmetric and symmetric exchange interactions between electric dipoles.** The symmetry operations are defined in Supplementary Table 21. In most cases, symmetry operations forbid some specific $A'_{ij,\alpha\beta}$ and/or $S'_{ij,\alpha\beta}$ components, making these components be zero (listed in the table). As for the symmetric exchange interaction, n_x symmetry operation ($n \geq 3$) further restricts that $S'_{ij,yy} = S'_{ij,zz}$ ($S'_{ij,yy} = S'_{ij,zz}$ can be non-zero).

Notations	A'_{ij}	S'_{ij}
$\bar{1}$	$A'_{ij,yz} = A'_{ij,xz} = A'_{ij,xy} = 0$	None
$\mathfrak{m}_x$	$A'_{ij,yz} = 0$	$S'_{ij,xz} = S'_{ij,xy} = 0$
$\mathfrak{m}_y$	$A'_{ij,yz} = A'_{ij,xy} = 0$	$S'_{ij,yz} = S'_{ij,xy} = 0$
$\mathfrak{m}_z$	$A'_{ij,yz} = A'_{ij,xz} = 0$	$S'_{ij,yz} = S'_{ij,xz} = 0$
2_y	$A'_{ij,xz} = 0$	$S'_{ij,yz} = S'_{ij,xy} = 0$
2_z	$A'_{ij,xy} = 0$	$S'_{ij,yz} = S'_{ij,xz} = 0$
$n_x(n \geq 2)$	$A'_{ij,xy} = A'_{ij,xz} = 0$	$n = 2$: $S'_{ij,xz} = S'_{ij,xy} = 0$<br>$n \geq 3$: $S'_{ij,yz} = S'_{ij,xz} = S'_{ij,xy} = 0$, $S'_{ij,yy} = S'_{ij,zz}$

SUPPLEMENTARY REFERENCES

[1] Dresselhaus, M. S., Dresselhaus, G. & Jorio, A. *Group Theory: Application to the Physics of Condensed Matter* (Springer Berlin, Heidelberg, 2007).

[2] Qi, Y. *et al.* Stabilization of competing ferroelectric phases of HfO_2 under epitaxial strain. *Phys. Rev. Lett.* **125**, 257603 (2020).

[3] Huan, T. D., Sharma, V., Rossetti, G. A. & Ramprasad, R. Pathways towards ferroelectricity in hafnia. *Phys. Rev. B.* **90**, 064111 (2014).

[4] Batra, R., Tran, H. D. & Ramprasad, R. Stabilization of metastable phases in hafnia owing to surface energy effects. *Appl. Phys. Lett.* **108**, 172902 (2016).

[5] Wang, J., Li, H. & Stevens, R. Hafnia and hafnia-toughened ceramics. *J. Mater. Sci.* **27**, 5397–5430 (1992).

[6] Curtis, C., Doney, L. & Johnson, J. Some properties of hafnium oxide, hafnium silicate, calcium hafnate, and hafnium carbide. *J. Am. Ceram. Soc.* **37**, 458–465 (1954).

[7] Ohtaka, O., Yamanaka, T. & Kume, S. Synthesis and X-ray structural analysis by the rietveld method of orthorhombic hafnia. *J. Ceram. Soc. Jpn.* **99**, 826–827 (1991).

[8] Liu, L.-G. New high pressure phases of ZrO_2 and HfO_2. *J. Phys. Chem. Solids* **41**, 331–334 (1980).

[9] Pathak, S., Mandal, G., Das, P. & Dey, A. B. Structural characteristics of HfO_2 under extreme conditions. *Mater. Chem. Phys.* **255**, 123633 (2020).

[10] Xu, X. *et al.* Kinetically stabilized ferroelectricity in bulk single-crystalline HfO_2: Y. *Nat. Mater.* **20**, 826–832 (2021).

[11] Hann, R. E., Suitch, P. R. & Pentecost, J. L. Monoclinic crystal structures of ZrO_2 and HfO_2 refined from X-ray powder diffraction data. *J. Am. Ceram. Soc.* **68**, C–285 (1985).

[12] Schroeder, U., Park, M. H., Mikolajick, T. & Hwang, C. S. The fundamentals and applications of ferroelectric HfO_2. *Nat. Rev. Mater.* **7**, 653–669 (2022).

[13] Zhao, H. J., Chen, P., Prosandeev, S., Artyukhin, S. & Bellaiche, L. Dzyaloshinskii–Moriya-like interaction in ferroelectrics and antiferroelectrics. *Nat. Mater.* **20**, 341–345 (2021).

[14] Moriya, T. Anisotropic superexchange interaction and weak ferromagnetism. *Phys. Rev.* **120**, 91 (1960).

[15] Dong, S., Xiang, H. & Dagotto, E. Magnetoelectricity in multiferroics: a theoretical perspective. *Natl. Sci. Rev.* **6**, 629–641 (2019).

[16] Chen, P., Zhao, H. J., Prosandeev, S., Artyukhin, S. & Bellaiche, L. Microscopic origin of the electric Dzyaloshinskii-Moriya interaction. *Phys. Rev. B.* **106**, 224101 (2022).
